# Differences in the Virulence Between Local Populations of *Puccinia striiformis* f. sp. *tritici* in Southwest China

**DOI:** 10.3390/plants13202902

**Published:** 2024-10-17

**Authors:** Fang Yang, Yunjing Wang, Zhiying Ji, Jiahui Liu, Mei Zhang, Yunliang Peng, Jie Zhao, Hongli Ji

**Affiliations:** 1 MoA Key Laboratory for Integrated Management of Pest on Crops in Southwest China/Institute of Plant Protection, Sichuan Academy of Agricultural Sciences, Chengdu 610066, China; yfwe928@163.com (F.Y.);; 2 College of Plant Protection, Northwest A&F University, Xianyang 712199, China; 3 Plant Protection Station, Sichuan Agricultural Bureau, Chengdu 610040, China

**Keywords:** wheat stripe rust, virulence, race, resistance expression, Southwest China

## Abstract

The virulence analysis of *Puccinia stiiformis* f. sp. *tritici* (*Pst*), the cause of wheat stripe rust, is essential for predicting and managing the disease epidemic in Southwest China, where the wheat cultivation has significantly reduced in the past few decades due to the impact of this disease. From 2020 to 2021, 196 *Pst* isolates were collected from Guizhou, Yunnan, and Sichuan. The virulence and race assessments were conducted using Chinese differential genotypes. Additionally, the resistance expression of 102 wheat lines was evaluated in 2021 in two disease nurseries located in Ningnan and Jiangyou. All the 45 *Pst* isolates from Guizhou and Yunnan belonged to pathogroup Hybrid 46, with 36 identified as race CYR32. Among the 69 isolates from the Liangshan Prefecture, 67 belonged to the Hybrid 46 group, while the remaining two were identified as race CYR34 in the G-22 group. Furthermore, all 79 isolates from the western Sichuan Basin belonged to the G-22 group, with 54 identified as race CYR34. The diversity indices of the *Pst* populations from Guizhou, Sichuan, and Yunnan exhibited a sequential decline. Virulence variation among the *Pst* populations from Yunnan, Guizhou, and the Ganzi-Liangshan region was minimal; however, significant virulence differences were observed when these populations were compared to those from the western Sichuan Basin. Results from disease nurseries indicated that *Pst* virulence was notably stronger in Ningnan compared to that in Jiangyou. The Sichuan Basin exhibits a notable diversity in *Pst* virulence, coupled with a more frequent genetic exchange occurring between the Liangshan Prefecture and the Yunnan-Guizhou Plateau. This information is essential for developing effective management strategies to mitigate the impact of wheat stripe rust in this region.

## 1. Introduction

*Puccinia stiiformis* is an obligate biotrophic species within the genus *Puccinia*, which belongs to the family Pucciniaceae under the order Uredinales of the class Urediniomycetes in phylum Basidiomycota [1]. Stripe rust disease caused by *Puccinia stiiformis* f. sp. *tritici* (*Pst*) is among the most devastating diseases of wheat in more than 60 countries [2,3,4,5]. Major stripe rust epidemics and severe yield losses have been reported in Asia [6,7,8,9], America [2], Europe [10], and Oceania [11,12]. Since the 1950s, China has experienced eight significant epidemics of stripe rust in 1950, 1964, 1983, 1985, 1990, 2002, 2017, and 2020, leading to a cumulative loss of over 13.8 million metric tons in wheat production [13]. In China, the wheat stripe rust is endemic to the cold, mountainous areas of high altitudes in the west and migrates eastward to the main areas of wheat production mainly located in the low-altitude reaches of Yangtze (/Chang), Huai, Yellow (/Huang), and Hai Rivers where the pathogen could not survive the hot summer [14]. According to Chen et al. [15], the epidemiological zones for wheat stripe rust in China were divided into three regions: the over-summering region, the winter reproductive region, and the spring epidemic region. Liangshan Yi and Ganzi Tibetan Autonomous Prefectures in Sichuan Province are the key over-summering regions, while the Sichuan Basin is one of the winter reproductive regions. In recent years, attention has expanded to *Pst* populations in Southwest China, such as Yunnan and Guizhou, and studies have indicated that the *Pst* population there could spread northeastward, reaching Sichuan and Hubei Provinces and even extending to the coastal areas, such as Shandong and Zhejiang [16,17,18,19,20,21].

Chinese races of *Pst* were coded with CYR (Chinese yellow rust) and differential names series with a relative frequency of more than 10% [22,23]. The designation of CYR series began with CYR1 in 1957 and progressed to the present CYR34, which was first detected in 2009 in Pi County of Sichuan Province and designated in 2016 [23,24]. CYR34 exhibits virulence to *Yr26* (=*Yr24*) and *Yr10*, which has rendered numerous wheat varieties with these resistance genes to be susceptible, such as ‘Guinong 22’, ‘Chuanmai 42’, and ‘Lantian 17’, contributing to a new round of wheat stripe rust epidemics in China [23]. In addition to CYR series, there are also pathotypes that are virulent to a specific genotype of Chinese differential sets, such as the Hybrid 46 (Hy46) pathotype, Suwon 11 (Su11) pathotype, Lovrin 10/13 (Lv10/13) pathotype, Guinong 22 (G22) pathotype, and Jubilejina 2 (Ju2) pathotype [25]. At present, the CYR34, CYR33, CYR32, and G22 pathotypes are the predominant races/pathotypes [26,27,28,29]. Moreover, new races virulent to ‘Zhong 4’ and *Yr5* detected in recent years pose a potential threat to wheat production [29,30,31].

Breeding and deploying resistant varieties are the most effective and environmentally sustainable approaches to managing wheat stripe rust [14,32]. To date, 86 *Yr* genes have been formally designated for stripe rust resistance (*Yr1* to *Yr86*); among them, only *Yr15*, *Yr45*, and *Yr61* are effective to prevalent Chinese *Pst* races [30,33,34,35,36]. It is commonly observed that the emergence of new *Pst* races could result in the breakdown of resistance in newly released wheat varieties within 3 to 5 years of their commercial cultivation [37,38]. Conducting a virulence assay on the *Pst* population is crucial for the breeding and deployment of resistant varieties, and it is vital for predicting and controlling wheat stripe rust [2,39]. The virulence profile of *Pst* populations is also an essential characteristic for analyzing the origins and migration patterns of these pathogens [40].

Sichuan Province, an important wheat-producing region in Southwest China, saw its wheat cultivation decrease to 588,780 hectares by 2022 [41]. This reduction was primarily due to recurring epidemics of stripe rust and other diseases, with the majority of the cultivation now concentrated in the northwestern part of the Sichuan Basin, around the cities of Mianyang, Deyang, and Guangyuan. Additionally, the Jinsha River area, which is part of the upper Yangtze River, is another important wheat-producing region in Sichuan, where wheat crops and their voluntary seedlings can be found annually at different altitudes [14]. In addition to the long-recognized source from Longnan area, the Yunnan-Guizhou Plateau has recently been identified as another significant source of *Pst* affecting the Sichuan Basin [14,42,43,44,45,46,47]. This report presents the findings from virulence assays conducted on *Pst* isolates collected from Southwest China, including the provinces of Yunnan, Guizhou, and Sichuan, during the wheat growing seasons of 2020 and 2021. The aim is to inform the deployment of resistant varieties and to analyze the potential sources of different *Pst* populations, thereby enhancing our understanding of their spread and impact on wheat production.

## 2. Results

### 2.1. The Composition of Pst Races and Virulence Types

**Figure 1:**
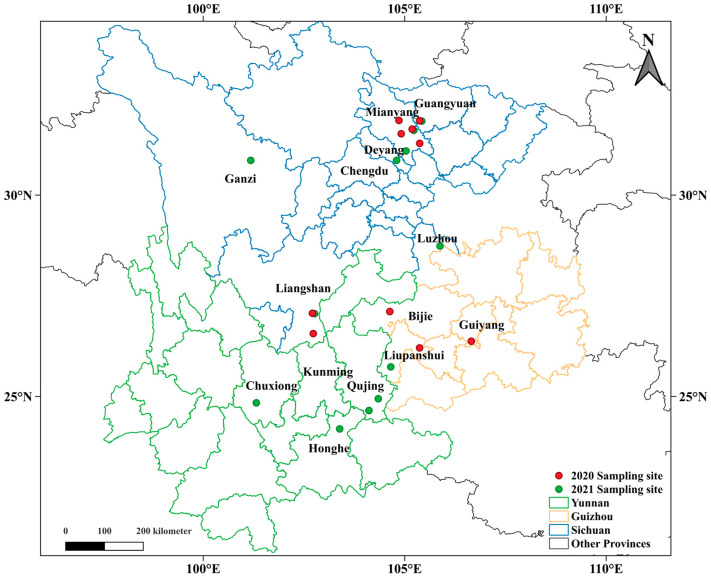
Sampling locations of *Puccinia stiiformis* f. sp. *tritici* (*Pst*)-infected leaves in the years 2020 and 2021. The red dots indicate the sampling sites in 2020, and the green dots indicate the sampling sites in 2021. The blue boundaries delineate the cities and prefectures of Sichuan Province, the green boundaries delineate the cities and prefectures of Yunnan Province, and the orange boundaries delineate the cities and prefectures of Guizhou Province.

The races and virulence types of *Pst* isolates collected from Yunnan, Guizhou, and Sichuan (Figure 1) are listed in Table 1 and Table 2. In Spring of 2020 and 2021, respectively, 7 and 5 isolates were collected from Guizhou Province (GZ). All 12 isolates were identified as pathogroup Hybrid 46, and 6 of them belonged to the race CYR32. In Yunnan Province (YN), 32 isolates were collected in the spring of 2021; all of them were in pathogroup Hybrid 46, and 30 of them belonged to race CYR32. There were big differences in the compositions of races and virulent types between samples collected from different regions in Sichuan. Respectively, 47 and 22 isolates were collected from Liangshan Prefecture (SC-L), located in the reach of the Jinsha River bordering Yunnan and Sichuan, in early spring of 2020 and 2021; 67 of them belonged to the Hybrid 46 group, 49 isolates were in race CYR32, while only 2 isolates were identified as CYR34 in G-22 group. Three isolates collected from Ganzi Prefecture (SC-G) in 2021 were all identified as CYR34. In early 2020, 43 isolates collected from the cities Guangyuan and Mianyang located in the northwest part of Sichuan Basin (SC-W) all belonged to the G-22 group, of which 29 isolates were identified as race CYR34. In early 2021, 17 isolates were collected from Mianyang; all these isolates belonged to the G-22 group, and 25 of them were identified as race CYR34. In 2021, one isolate collected from Luzhou City, located in the southeast part of Sichuan Basin (SC-E), also belonged to the G-22 group.

**Figure 2:**
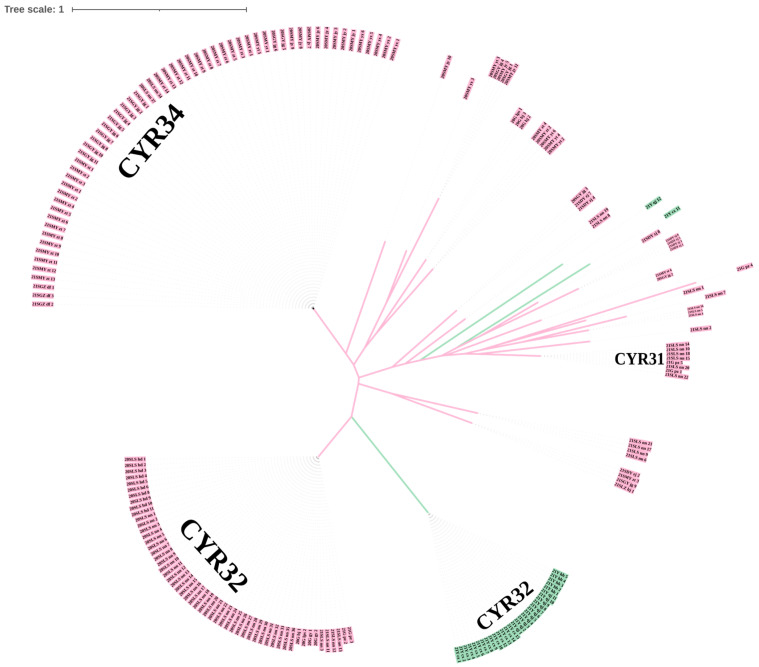
Phylogenetic tree of *P. striiformis* f. sp. *tritici* isolates collected in different regions of Southwest China based on the virulence data in 2020 and 2021. The isolates highlighted in pink were virulent to ‘Chuanmai 104’, and the isolates highlighted in green were avirulent to ‘Chuanmai 104’. Isolate codes begin with “20” or “21” indicate the sampling years 2020 and 2021, respectively. The letters “S”, “G”, and “Y” represent the provinces: Sichuan, Guizhou, and Yunnan, respectively. The capital letters that follow “20S” or “21S” represent cities in Sichuan, “MY” for Mianyang City, “GY” for Guangyuan City, “DY” for Deyang City, “LZ” for Luzhou City, “LS” for Liangshan Yi Autonomous Prefecture, and “GZ” for Ganzi Tibetan Autonomous Prefecture; the lowercase letters that follow city names represent counties, districts, or cities belonging to the city: “yx” for Youxian District, “jy” for Jiangyou City, “yt” for Yanting County, “zt” for Zitong County, “st” for Santai County, “zj” for Zhongjiang County, “jg” for Jiange County, “hj” for Hejiang County, “hd” for Huidong County, “nn” for Ningnan County, and “df” for Daofu County. The lowercase letters that follow “20G” or “21G” represent cities in Guizhou: “pz” for Panzhou City; “lps” for Liupanshui City, “gy” for Guiyang City, and “hz” for Hezhang County. The lowercase letters that follow “21Y” represent cities in Yunnan: “cx” for Chuxiong Yi Autonomous Prefecture, “hh” for Honghe Hani and Yi Autonomous Prefecture, and “qj” for Qujing City.

Based on the virulence phenotypes of the isolates to the Chinese differential genotypes, cluster analysis of the isolates was conducted (Figure 2). When the virulence to ‘Chuanmai 104’, a wheat variety widely planted in Sichuan, was considered, the tested 196 isolates could be clustered into different groups. The CYR32 race was divided into two groups. All the CYR32 isolates from Liangshan Prefecture and Guizhou Province were virulent to ‘Chuanmai 104’, while those from different regions in Yunnan Province were avirulent to ‘Chuanmai 104’. All the isolates of the CYR34 race were virulent to ‘Chuanmai 104’. All the 12 isolates from Guizhou were virulent to ‘Chuanmai 104’, while no virulent isolates to such variety were found among the 32 isolates from Yunnan Province. All the 152 isolates from Sichuan were virulent to ‘Chuanmai 104’ regardless of their geographic origin.

### 2.2. Virulence Frequencies

**Figure 3:**
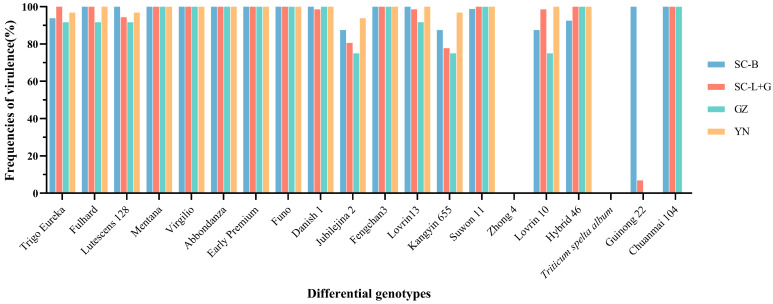
Virulence frequency of *Pst* isolates from Southwest China in 2020 and 2021 to the 19 Chinese differential genotypes and the supplemental differential genotype ‘Chuanmai 104’. SC-B, Sichuan Basin; SC-L+G, Liangshan Prefecture and Ganzi Prefecture, Sichuan Province; YN, Yunnan Province; GZ, Guizhou Province.

Analyzing the virulence frequencies of *Pst* isolates against the 19 Chinese differential genotypes (Figure 3), it can be seen that all isolates from Southwest China showed avirulence to ‘Zhong 4’ and *Triticum spelta album*. Isolates from Yunnan and Guizhou were avirulent to ‘Guinong 22’, and isolates from the Liangshan and Ganzi areas of Sichuan exhibited a virulence frequency below 10% to ‘Guinong 22’, contrasting with the isolates from the Sichuan Basin whose virulence frequency reached 100%. The virulence frequencies of all *Pst* isolates to the remaining 16 Chinese differential genotypes exceeded 75%.

### 2.3. Virulence Diversity and Population Differentiation

The virulence diversity of each *Pst* population from different regions in Southwest China is shown in Table 3. The *Pst* population from Guizhou has given rise to the highest virulence diversity. In Guizhou, the Nei’s diversity index (*Hs*) was 0.090, the Simpson index (*Si*) was 0.681, the Shannon normalized index (*Sh*) was 0.546, and the Kosman index (*KWm*) was 0.111. The population in Yunnan demonstrated the lowest diversity according to the indices with *Hs* at 0.014, *Si* at 0.119, *Sh* at 0.252, and *KWm* at 0.015. The diversity indices of the *Pst* populations from Sichuan Basin, Ganzi, and Liangshan were in between.

Nei’s genetic identity between the different *Pst* populations ranged from 0.9517 to 0.9972, and Nei’s genetic distance varied from 0.0028 to 0.0495 (Table 4). Nei’s genetic distance between *Pst* populations from YN (Yunnan) and SC-L+G (Liangshan and Ganzi of Sichuan) was the closest among the four populations, which was 0.0028, followed by the distance between the populations of SC-L+G and GZ (Guizhou) (0.0039) and then the distance between the populations of YN and GZ (0.0079). Nei’s genetic distances between the populations from SC (Sichuan Basin) and SC-L+G, GZ, and YN were, respectively, 0.0440, 0.0452, and 0.0495, indicating the larger identity among SC-L+G, YN, and GZ populations as with the identity values of 0.9972, 0.9962, and 0.9922. Interestingly, the genetic distance between *Pst* populations from two regions in Sichuan Province was much larger than that between *Pst* populations from Ganzi-Liangshan area and Yunnan or Guizhou. Correspondingly, the genetic identity of Ganzi-Liangshan area and the Sichuan Basin is 0.9570, lower than the genetic identity of Ganzi-Liangshan and Yunnan (0.9972) or Guizhou (0.9962).

**Figure 4:**
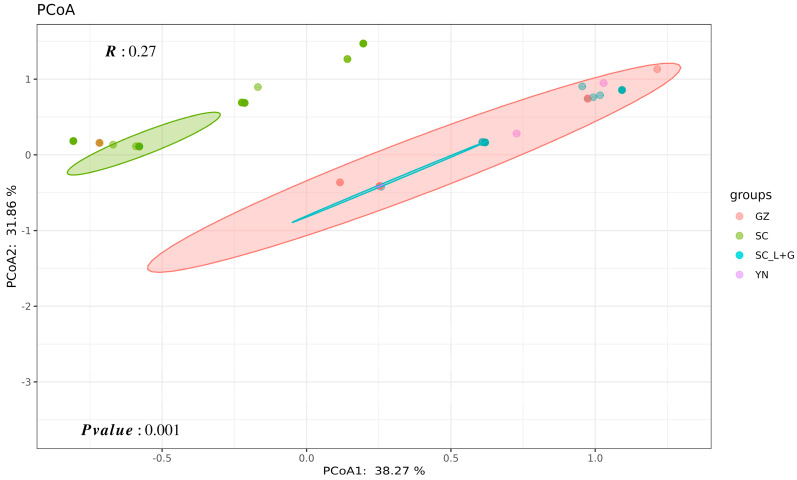
Principal coordinate analysis (PCoA) of the *Pst* virulence from Sichuan Basin (SC), Ganzi-Liangshan areas of Sichuan (SC_L+G), Yunnan (YN), and Guizhou (GZ).

The results of principal coordinate analysis (PCoA) indicated that the PCoA1 could explain 38.27% of the virulence variation, while PCoA2 could explain 31.86% of the virulence variation (Figure 4). The isolates of each population were largely distributed in clusters, of which the most *Pst* populations from the Yunnan, Guizhou, as well as Ganzi-Liangshan areas were clustered together. The isolates from Sichuan Basin were separated in different clusters.

### 2.4. The Virulence of Pst Populations to Wheat Varieties and Lines at Ningnan and Jiangyou

In the autumn of 2020, 102 wheat varieties and lines with different resistance genotypes against stripe rust were planted in two disease nurseries located in Ningnan and Jiangyou. The two nurseries are situated in the Jinsha River valley of the Liangshan Prefecture and the northwestern part of Sichuan Basin, respectively. The infection type, average severity, and leaf incidence of stripe rust were investigated and recorded at the milky stage of wheat (Table 5). In Ningnan and Jiangyou, 46 and 33 wheat varieties or lines were susceptible to the stripe rust, respectively, with 3 to 4 infection types. Among the 24 wheat lines known to carry resistance genes, 16 lines in Ningnan displayed susceptible disease symptoms, while 14 lines in Jiangyou showed immunity or resistance to stripe rust. It is noteworthy that *Triticum spelta album*, believed to bear *Yr5*, was found to be susceptible in Ningnan but resistant in Jiangyou. Another line, Mingxian 169*6/*Yr5*, which also carries *Yr5*, remained immune to the disease in both sites.

## 3. Discussion

Several studies have focused on the *Pst* population structure of Southwest China and found that *Pst* populations in the Sichuan Basin frequently exchange genes with other areas. For example, *Pst* populations in the northwest of the Sichuan Basin could originate from Longnan in Gansu Province, and in the south, they may originate from the Yunnan-Guizhou Plateau or even the Tibetan Plateau [14,23,24,25,26,42,43,44,45,46,47]. In this study, we assessed the virulence of 196 *Pst* isolates from Sichuan, Yunnan, and Guizhou Provinces during 2020–2021. All isolates collected from the northwest part of the Sichuan Basin were identified as the pathogroup G-22, with CYR34 as the predominant race. All isolates from Yunnan and Guizhou were identified as the Hybrid 46 group, with CYR32 being the predominant race. The three isolates from the Ganzi Prefecture in 2021 were identified as CYR34. Most isolates from the Liangshan Prefecture of Sichuan were identified as the Hybrid 46 pathogroup, with a small number of the G-22 group. These results indicate that the physiological races in the Yunnan-Guizhou Plateau and the adjacent Liangshan Prefecture of Sichuan share higher similarities in their composition, while the northwest part of the Sichuan Basin exhibits significant differences in its physiological race composition compared to these areas. Nei’s genetic distance and the principal coordinate analysis (PCoA) results show that the distances between the Yunnan, Guizhou, and Liangshan-Ganzi populations are relatively close, while the northwest Sichuan Basin population is more distant from these three populations. Such results clearly indicate that the *Pst* population from the Yunnan-Guizhou Plateau has little impact on the northwest part of the Sichuan Basin, and these results are consistent with a recent study which found that the *Pst* populations in Guizhou are genetically similar to and closely distributed with those in Yunnan and Liangshan, while the population in the Sichuan Basin exhibits a relatively independent genetic structure with less genetic interchange [48].

There has been no evidence that *Pst* can stably survive over summer in Guizhou Province, although sporadic diseased plants have been witnessed in some cold and wet summers. In this research, we found the identity between the *Pst* populations from the Liangshan Prefecture and Guizhou. Considering that the sowing time can be as early as in late September, the first incidence of disease can be as early as late October along the Jinsha River in the Liangshan Prefecture [49], along with its short distance to the alpine wheat cultivation areas in the Liangshan and Ganzi Prefectures, and we speculate that the wheat cultivation area along the Jinsha River could be the important area to bridge the disease epidemic in Guizhou and the alpine areas in the further north and west. Further studies on the resistance expression in wheat hosts, molecular and virulence characterization of more *Pst* populations, as well as the analysis of wind patterns are necessary to identify the source and transmission routes of the stripe rust fungus in all of Southwest China.

Some studies have reported that the diversity of the *Pst* population in the Sichuan Basin was higher than that in both Yunnan and Guizhou [19,48]. Similarly, Jiang et al. found that the diversity in Guizhou and most regions of Yunnan was lower than that in areas containing over-summering regions [18]. In contrast, other studies have suggested that the *Pst* population from Yunnan possesses higher genetic diversity compared to that from Sichuan [17,20,50]. In this research, we found that the virulence diversities of the populations from Guizhou and Yunnan are, respectively, the highest and the lowest, with those from Liangshan-Ganzi and the northwest part of the Sichuan Basin falling in between. Variations in diversity results in different studies may be due to the differences in times, ranges, and methods of sampling and evaluation standards. A systematic sampling from all wheat production in Southwest China at different stages of disease epidemic is needed for the exact evaluation.

The results of the laboratory identification of race and virulence types in this research indicated that the virulence of *Pst* populations in Liangshan Prefecture, Guizhou, and Yunnan Province was weaker than that in the northwest of the Sichuan Basin. However, the resistance expression of the same set of wheat varieties and lines in the disease nurseries in Ningnan and Jiangyou, respectively, along the Jinsha River in the Liangshan Prefecture and Mianyang City in the northwest of the Sichuan Basin, suggested a stronger virulence of *Pst* in the Jinsha River reach. These contradictory findings by virulence assay using different methods highlights the demands for more supplementary wheat lines during laboratory virulence assay in order to precisely analyze the local *Pst* population. For example, we have observed differences in virulence to ‘Chuanmai 104’ between the CYR32 isolates from Yunnan and Guizhou in this research, which reveals that race CYR32 in the two regions has experienced genetic differentiation, although the reason for such differentiation has to be studied.

Currently, the disease nurseries to evaluate the resistance to *Pst* in Sichuan Province were located in the west part of the Sichuan Basin. Due to the stronger virulence indicated by the more susceptible wheat varieties in the disease nurseries in Ningnan than that in Jiangyou and potential effects of the *Pst* population along the Jinsha River onto Guizhou, and further onto southern and eastern part of Sichuan Basin, the resistance evaluation at the Ningnan nursery is important to the successful breeding and deployment of resistant varieties both in Guizhou and Sichuan. Obviously, more disease nurseries in the southern and eastern parts of the Sichuan Basin are also necessary.

## 4. Materials and Methods

### 4.1. Field Survey and Isolate Collection

Regular field surveys were conducted annually across wheat growing areas in Southwest of China from late January to early April during 2020 to 2021. Infected wheat leaves were collected from disease nurseries and commercial wheat fields (Figure 1). Date of collection, location, cultivar, severity, collector, and any other relevant information was recorded for each sample whenever possible (Supplementary Table S1). Diseased leaves were air-dried and dispatched in paper envelopes to the lab in Chengdu and stored at 4 to 5 °C until spores were collected for inoculation and multiplication. A total of 196 stripe rust samples were collected from different regions in Sichuan, Guizhou, and Yunnan Province during 2020 and 2021 (Supplementary Table S1).

### 4.2. Disease Nurseries

Disease nurseries contained 102 wheat cultivars, including important resistant materials, differentials, and near-isogenic *Yr* gene lines [40]. They were planted at two locations (see Table 5) in Sichuan, respectively, in Ningnan County at the riverside of the Jinsha River and Jiangyou City in the northwest part of the Sichuan Basin during the local growth season of wheat to determine the effectiveness of resistance. At the late milky stage, average severity (%, the percentage of infected tissue), incidence (%, the percentage of infected leaves), and infection type were recorded using the modified Cobb scale [51].

### 4.3. Identification of Race and Virulence

Flat, clean leaves with typical stripe rust lesions were selected, and surface impurities were rinsed off using tap water. The leaves were placed with the front side up in a Petri dish lined with moist filter paper. A fine mist of water was gently sprayed onto the leaves to promote the germination of stripe rust spores. The Petri dish was then placed in a dark, cool (4 °C) environment for 8–12 h to maintain humidity. Once the first leaf of the susceptible variety ‘Mingxian 169’ was fully unfolded, an inoculation needle was used to carefully pick individual uredinial pustules from the leaf sample and gently smear them in the middle area of the wheat leaf, either from top to bottom or bottom to top. After inoculation, the seedlings were covered with a transparent plastic cylinder and placed in a dark, humid environment at 9–13 °C for 24 h. The seedlings were then transferred to a greenhouse rack at 14–19 °C to allow disease incubation under 10 h of light per day. Fifteen days after inoculation, urediniospores from each isolate were collected into 1.5 mL centrifuge tubes and diluted with 7100 Engineered Fluid (3M, MN, USA) until the suspension appeared pale yellow. The diluted spore suspension was evenly applied to ‘Mingxian 169’ leaves using a pipette for isolate propagation. After inoculation, water mist was sprayed to maintain humidity, and the wheat seedlings were covered with transparent plastic cylinders, with the humidity and incubation conditions remaining the same.

Once the ‘Mingxian 169’ seedling above had fully developed the disease, the wiping method was used to inoculate 19 Chinese differential genotypes, the susceptible variety ‘Mingxian 169’, and the Sichuan supplementary differential genotype ‘Chuanmai 104’. Sterilized peat and soil mixture was placed in 20 cm × 30 cm enamel trays, with two groups of differential genotypes sown in each tray. Each row contained five varieties, and each variety was sown with 4–6 seeds and grown in a rust-free greenhouse. About 10 days after sowing, when the first leaf had fully expanded, the trays were placed in a large box, and wheat leaves with propagated urediniospores were used to wipe and contact the differential genotype seedlings from different angles. After inoculation, a water mist was sprayed to maintain humidity, and the wheat plants were transferred to a humidity chamber for 24 h. Afterward, they were moved to a greenhouse under the same temperature and light conditions. Fifteen to eighteen days after inoculation, once the susceptible control had fully developed the disease, the infection type (IT) data was recorded. Races of *P. striiformis* f. sp. *tritici* were determined by IT based on the 0 to 4 scale [52], i.e., no visible symptoms (IT 0), necrotic or chlorotic flecks (IT 0;), necrotic or chlorotic blotches with very low level of sporulation (IT 1), moderate uredinia with necrosis/chlorosis (IT 2), moderate-to-abundant uredinia with chlorosis (IT 3), and abundant uredinia without necrosis and chlorosis (IT 4). Infection types 0 to 2 were considered as avirulent, and 3 to 4 to be virulent [40]. Based on the reactions of different physiological races and virulence types to each of the Chinese differential genotypes according to the Agricultural Industry Standard of China (NY/T 1443.1-2007) [53], the virulence types of the identified isolates can be determined.

### 4.4. Cluster Analysis and Construction of Phylogenetic Trees

In order to intuitively see the classification of isolates from different regions, cluster analysis was performed using the data of virulence identification. Infection types 0–2 were recorded as “0” and infection types 3–4 were recorded as “1”; then, the results were transformed by this rule to obtain matrix data. Perform cluster analysis on this matrix data using the *ape* package of R (version 5.7-0) and *ggtree* package of R (version 3.10.0), construct a phylogenetic tree using the Neighbor-Joining method, and output a file in nwk format. The nwk file was placed on the online software iTOL (https://itol.embl.de/ (accessed on 19 June 2024)) to set and output the image format.

### 4.5. Race Diversity Measurements

Frequencies of races within the population collected during different years or diverse geographical regions were compared. For diversity analysis, the resistance (R) and sensitivity (S) reactions of each isolate to the differentiating varieties were first transformed, respectively, to “0” and “1” values. Then the Nei diversity index (*Hs*), Shannon normalized index (*Sh*), Shannon index (*SH*), Simpson index (*Si*), Kosman index (*KWm*), Stoddart index (*St*), Evenness (*E*), and Gleason index of richness (*G*) were calculated using Virulence Analysis Tool (VAT) online software at https://en-lifesci.tau.ac.il/node/3094 (accessed on 20 April 2024) to evaluate the gene or genotype diversity of different populations (Table 3). Unweighted pair group method with arithmetic mean (UPGMA) in NTSYSpc software (version 2.11) was used to calculate Nei’s genetic identity and distance in order to analyze the similarity between different *Pst* populations. Principal coordinate analysis (PCoA) was conducted using online tool at https://hiplot.com.cn (accessed on 20 April 2024).

## Figures and Tables

**Table 1:** The virulence composition of *Puccinia stiiformis* f. sp. *tritici* (*Pst*) from different populations of Southwest China in 2020.

Pathogroup	*Pst* Race/Virulence Type	Total Number of Isolates	Number of Isolates Per Pathogroup	SC-W *		SC-L	GZ
				Mianyang	Guangyuan		
Hybrid 46 group	CYR32	49	52	0	0	45	4
	HY_108	3		0	0	0	3
G-22 group	CYR34	31	45	27	2	2	0
	G22_014	1		1	0	0	0
	G22_083	1		0	1	0	0
	G22_104	5		5	0	0	0
	G22_183	1		0	1	0	0
	G22-244	5		3	2	0	0
	G22_245	1		1	0	0	0
Total number of isolates		97	97	43		47	7

* SC-W, Western Sichuan Basin, including counties, districts and cities belonging to Mianyang City (Youxian District, Jiangyou City, Yanting County, Zitong County) and Guangyuan City (Jiange County); SC-L, Liangshan Yi Autonomous Prefecture, Sichuan Province, including Huidong County and Ningnan County; GZ, Guizhou Province, including Liupanshui City, Guiyang City and Hezhang County.

**Table 2:** The virulence composition of *P. stiiformis* f. sp. *tritici* from different populations of Southwest China in 2021.

Pathogroup	*Pst* Race or/Virulence Type	Total Number of Isolates	Number of Isolates Per Pathogroup	SC-W *			SC-E	SC-L	SC-G	GZ	YN
				Mianyang	Deyang	Guangyuan	Luzhou				
Hybrid 46 group	CYR31	8	59	0	0	0	0	6	0	2	0
	CYR32	36		0	0	0	0	4	0	2	30
	HY_008_1	4		0	0	0	0	4	0	0	0
	HY_019	2		0	0	0	0	2	0	0	0
	HY_035	1		0	0	0	0	0	0	0	1
	HY_183	1		0	0	0	0	1	0	0	0
	HY_311	3		0	0	0	0	3	0	0	0
	Other HY	4		0	0	0	0	2	0	1	1
G-22 group	CYR34	28	40	15	0	10	0	0	3	0	0
	G22_083	2		0	2	0	0	0	0	0	0
	G22_108	4		1	1	1	1	0	0	0	0
	G22_183	1		1	0	0	0	0	0	0	0
	G22-431	1		0	1	0	0	0	0	0	0
	Other G-22	4		0	4	0	0	0	0	0	0
Total number of isolates		99	99	36			1	22	3	5	32

* SC-W, Western Sichuan Basin, including counties, districts and cities belonging to Mianyang City (Santai County, Zitong County), Deyang City (Zhongjiang County), and Guangyuan City (Jiange County); SC-E, Eastern Sichuan Basin, including counties, districts, and cities belonging to Luzhou City (Hejiang County); SC-L, Liangshan Yi Autonomous Prefecture (Ningnan County), Sichuan Province; SC-G, Ganzi Tibetan Autonomous Prefecture (Daofu County), Sichuan Province; GZ, Guizhou Province, including Panzhou City; YN, Yunnan Province, including Chuxiong Yi Autonomous Prefecture, Honghe Hani and Yi Autonomous Prefecture, and Qujing City.

**Table 3:** Virulence diversity of *P. striiformis* f. sp. *tritici* from different regions in Southwest China.

Parameter	SC *	SC_L+G	YN	GZ
Number of isolates	80	72	32	12
Nei’s diversity index, *Hs*	0.047	0.047	0.014	0.090
Simpson index, *Si*	0.562	0.532	0.119	0.681
Shannon normalized index, *Sh*	0.319	0.298	0.080	0.546
Kosman index, *KWm*	0.052	0.056	0.015	0.111
Stoddart index (*St*)	2.281	2.136	1.135	3.130
Shannon index (*SH*)	1.398	1.281	0.277	1.358
Evenness (*E*)	0.583	0.556	0.252	0.844
Gleason index of richness (*G*)	2.282	2.098	0.577	1.610

*: SC, Sichuan Basin; SC_L+G, Liangshan Prefecture and Ganzi Prefecture, Sichuan Province; YN, Yunnan Province, GZ, Guizhou Province.

**Table 4:** Nei’s genetic identity and genetic distance between populations of *P. striiformis* f. sp. *tritici* from different regions in Southwest China.

Populations	SC	SC_L+G	YN	GZ
SC		0.9570 *	0.9517	0.9558
SC_L+G	0.0440		0.9972	0.9962
YN	0.0495	0.0028		0.9922
GZ	0.0452	0.0039	0.0079	

* Nei’s genetic identity (right upper diagonal) and genetic distance (left lower diagonal). SC, Sichuan Basin; SC_L+G, Liangshan Prefecture and Ganzi Prefecture, Sichuan Province; YN, Yunnan Province, GZ, Guizhou Province.

**Table 5:** Resistance expression of different wheat varieties and lines in disease nurseries in Ningnan and Jiangyou in 2021.

Wheat Genotype/Cultivar	Gene	Ningnan *			Jiangyou		
		Infection Type	Severity (%)	Incidence (%)	Infection Type	Severity (%)	Incidence (%)
Heines VII	*Yr2*	3	30	10	2	100	30
Heies Peko	*Yr2.6*	3	5	5	2	90	70
Nord Desprez	*Yr3*+	3	60	10	2	100	60
Hybrid46	*YR4.3b*	3	5	5	0	0	0
Mingxian 169*6/*Yr5*	*Yr5*	0	0	0	0	0	0
*Triticum spelta album*	*Yr5*	3−	10	5	0	0	0
Reichersberg42	*Yr7*+	3	5	5	2	5	10
Compare	*Yr8.19*	3	70	40	2	100	70
Lovrin 10	*Yr9*	3−	10	30	4	90	100
Avocet S*6/*Yr9*	*Yr9*	0	0	0	2−	90	5
Lovrin 13	*Yr9*+	3	20	10	4	90	100
Mingxian 169*6/*Yr10*	*Yr10*	3	70	10	4	100	100
Joss Cambier	*Yr11*	0	0	0	3	100	80
Mega	*Yr12*	3	30	5	2	70	70
Hobbit	*Yr14*	3	40	5	3	80	60
Avocet S*6/*Yr15*	*Yr15*	3	50	20	2	100	70
Jupateco R *Yr18*	*Yr18*	1+	10	30	2	100	80
Avocet S*6/*Yr24*	*Yr24*	3	60	20	4	100	100
Avocet S*6/*Yr26*	*Yr26*	4	70	90	4	100	100
Carsten V	*Yr32*	3	40	10	2	100	60
C591	*YrC591*	2	20	10	2	80	10
Kangyin 655	*YrKy*	3	30	10	4	100	100
Spaldings Prolific	*YrSpP*	3	30	20	3	100	70
Suwon 11	*YrSu*	3−	5	20	4	100	100
Changmai 26		2	20	10	2	100	60
Changmai 29		2+	60	20	3	100	100
Changmai 32		2	60	70	2+	100	70
Changmai 33		2	50	40	2+	100	60
Changmai 34		2	30	30	3−	100	70
Changmai 35		2−	5	5	2	100	60
Chuanmai 42		2+	60	70	2−	5	1
Chuanmai 55		2	60	60	0	0	0
Chuanmai 58		2	60	10	0	0	0
Chuanmai 61		2+	30	20	0	0	0
Chuanmai 62		2	20	10	0	0	0
Chuanmai 82		2	20	10	2	10	1
Chuanmai 83		3	70	40	0	0	0
Chuanmai 86		2−	5	10	0	0	0
Chuanmai 93		2	10	20	0	0	0
Chuanmai 98		0	0	0	0	0	0
Chuanmai 104		2+	60	60	2−	5	1
Chuanmai 107		3	70	70	4	90	90
Chuanmai 602		2+	60	20	0	0	0
Chuanmai 604		2	20	5	0	0	0
Chuanmai 1131		3	70	90	0	0	0
Chuanmai 1145		4	90	90	3	60	60
Chuanmai 1247		2+	20	30	0	0	0
Chuanmai 1456		0;	5	5	0	0	0
Chuanmai 1546		3	70	60	0	0	0
Chuanmai 1557		2	20	30	0	0	0
Chuanmai 1580		2	30	30	0	0	0
Chuanmai 1826		2+	70	80	0	0	0
Chuannong 19		3−	60	20	3	100	90
Chuannong 26		2+	60	40	4	100	90
Chuannong 27		0	0	0	3	90	90
Chuannong 27		2+	30	30	3−	90	50
Chuannong 29		2	30	30	0	0	0
Chuannong 30		2+	70	60	3−	100	60
Chuannong 32		2	40	30	2	100	50
Chuanyu 23		3−	40	10	2	90	40
Fan 6		3−	60	20	4	100	100
Jianongmai 809		2	20	10	3−	90	80
Jimai 22		3−	60	40	4	100	100
Jimai 38		4	70	60	4	100	100
Jintai 170		4	80	70	4	100	100
Kechengmai 4		3	70	50	0	0	0
Kechengmai 5		2	40	10	2	100	50
Kechengmai 6		2	20	10	0	0	0
Lantian 19		0	0	0	0;	10	1
Lantian 26		0	0	0	0	0	0
Lantian 31		0	0	0	1	5	1
Little Clup		3−	80	90	4	90	100
Longjian 386		0	0	0	0	0	0
Lumai 23		3	30	10	4	100	100
Mianmai 37		4	70	80	0	0	0
Mianmai 46		4	70	80	3	90	70
Mianmai 51		2+	60	30	0	0	0
Mianmai 112		4	90	90	0	0	0
Mianmai 228		3	80	90	0	0	0
Mianmai 285		3	60	60	0	0	0
Mianmai 312		2+	20	10	0	0	0
Mianmai 367		2+	50	20	0	0	0
Mianmai 827		2	20	10	0	0	0
Mianmai 902		2	10	20	2	100	40
Mianmai 1501		2	20	10	0	0	0
Mianyang 11		4	90	100	4	100	100
Mianzimai 830		2	20	20	3−	100	80
Nanmai 618		3	60	20	2	5	5
Nanmai 660		2+	20	20	0	0	0
Nanmai 991		2−	5	20	3	10	20
Neimai 11		4	90	90	0	0	0
Neimai 316		2+	40	60	0	0	0
Neimai 366		2+	60	20	0	0	0
Neimai 836		3	90	90	0	0	0
Neimai 9		3	80	70	0	0	0
Neimai 9		3	70	70	0	0	0
Shi 4185		4	70	70	4	100	100
Tianxuan 50		0	0	0	1	40	5
Yangmai158		4	80	70	3	100	70
Yannong 15		2+	40	10	4	100	100
Zhong 4		0	0	0	0	0	0
Zhoumai 22		0	0	0	2	40	40

* The Ningnan nursery was located in Heinigou Village, Jingxing Town, Ningnan County, Liangshan Yi Autonomous Prefecture, Sichuan Province (27°2′15.5″ N, 102°45′49.2″ E, 1008 m asl); the Jiangyou nursery was located in Yangtingba Village, Wudu County, Jiangyou City, Mianyang City, Sichuan Province (31°53′46.5″ N, 104°47′17.6″ E, 530 m asl).

## Data Availability

The data presented in this study are available within the article and Supplementary Tables.

## Supplementary Materials

The following supporting information can be downloaded at: https://www.mdpi.com/article/10.3390/plants13202902/s1, Supplementary Table S1: The sampling information of diseased leaves infected by *Puccinia striiformis* f. sp. *tritici* in 2020 and 2021 for the race and virulence assay; Supplementary Table S2. The virulence and avirulence patterns of isolates (*Puccinia striiformis* f. sp. *tritici*) collected in 2020–2021 on the 21 differential genotypes.
