# Supplementary material for: Differences in the Virulence Between Local Populations of Puccinia striiformis f. sp. tritici in Southwest China
Source: Plants (Basel). 2024 Oct 17;13(20):2902. doi: 10.3390/plants13202902 (PMC11511155; doi:10.3390/plants13202902)
Supplement: Supplementary file 1 [file plants-13-02902-s001.zip › Supplementary Table/Table S1.pdf]

Supplementary Table S1. The sampling information of diseased leaves infected by *Puccinia striiformis* f. sp. *tritici* in 2020 and 2021 for the race and virulence assay.

**Isolates**	**Sampling location**	**Latitude**	**Longitude**	**Altitude(m)**	**Sampling Date**	**Varieties**
20SMY_yx_1	Zhulin Village, Jiangyou City, Mianyang City, Sichuan Province	31.744722	104.697778	478	2020.1.13-2.3	Unknown
20SMY_yx_2	Youxian District, Mianyang City, Sichuan Province				2020.1.13-2.3	Unknown
20SMY_yx_3	Youxian District, Mianyang City, Sichuan Province				2020.1.13-2.3	Unknown
20SMY_yx_4	Youxian District, Mianyang City, Sichuan Province				2020.1.13-2.3	Unknown
20SMY_yx_5	Youxian District, Mianyang City, Sichuan Province				2020.1.13-2.3	Unknown
20SMY_yx_6	Youxian District, Mianyang City, Sichuan Province				2020.1.13-2.3	Unknown
20SMY_jy_1	Yangtingba Village, Wudu Town, Jiangyou City, Mianyang City, Sichuan Province	31.8962479	104.7882409	529.6	2020.2.27-3.3	Unknown
20SMY_jy_2	Yangtingba Village, Wudu Town, Jiangyou City, Mianyang City, Sichuan Province	31.8962479	104.7882409	529.6	2020.2.27-3.3	Unknown
20SMY_jy_3	Yangtingba Village, Wudu Town, Jiangyou City, Mianyang City, Sichuan Province	31.8962479	104.7882409	529.6	2020.2.27-3.3	Unknown
20SMY_jy_4	Yangtingba Village, Wudu Town, Jiangyou City, Mianyang City, Sichuan Province	31.8962479	104.7882409	529.6	2020.2.27-3.3	Unknown
20SMY_jy_5	Yangtingba Village, Wudu Town, Jiangyou City, Mianyang City, Sichuan Province	31.8962479	104.7882409	529.6	2020.2.27-3.3	Unknown
20SMY_jy_6	Anping Village, Wudu Town, Jiangyou City, Mianyang City, Sichuan Province	31.808	104.7821	529	2020.2.27-3.3	Unknown
20SMY_jy_7	Anping Village, Wudu Town, Jiangyou City, Mianyang City, Sichuan Province	31.808	104.7821	529	2020.2.27-3.3	Unknown
20SMY_jy_8	Qingping Village, Guanshan Town, Jiangyou City, Mianyang City, Sichuan Province	31.767314	104.841478	559	2020.2.27-3.3	Unknown
20SMY_jy_9	Qingping Village, Guanshan Town, Jiangyou City, Mianyang City, Sichuan Province	31.767314	104.841478	559	2020.2.27-3.3	Unknown

20SMY_jy_10	Qingping Village, Guanshan Town, Jiangyou City, Mianyang City, Sichuan Province	31.767314	104.841478	559	2020.2.27-3.3	Unknown
20SMY_jy_11	Qingping Village, Guanshan Town, Jiangyou City, Mianyang City, Sichuan Province	31.767314	104.841478	559	2020.2.27-3.3	Unknown
20SGY_jg_1	Youai Village, Kiafeng Town, Jiange County, Guangyuan City, Sichuan Province	31.777	105.403	566	2020 2.28-3.9	Unknown
20SGY_jg_2	Zhongying Village, Kiafeng Town, Jiange County, Guangyuan City, Sichuan Province	31.81	105.399	550	2020 2.28-3.9	Unknown
20SGY_jg_3	Zhongying Village, Kiafeng Town, Jiange County, Guangyuan City, Sichuan Province	31.81	105.399	550	2020 2.28-3.9	Unknown
20SGY_jg_4	Yongsheng Village, Kiafeng Town, Jiange County, Guangyuan City, Sichuan Province	31.811	105.389	463	2020 2.28-3.9	Unknown
20SGY_jg_5	Youai Village, Kiafeng Town, Jiange County, Guangyuan City, Sichuan Province	31.777	105.403	566	2020 2.28-3.9	Unknown
20SGY_jg_6	Foergou Village, Liugou Town, Jiange County, Guangyuan City, Sichuan Province	31.98111	105.3623	544	2020 2.28-3.9	Unknown
20SMY_yt_1	Yanting County, Mianyang City, Sichuan Province				2020.2.29-3.29	Mingxian 169
20SMY_yt_2	Yanting County, Mianyang City, Sichuan Province				2020.2.29-3.29	Chuanyu 18
20SMY_yt_3	Yanting County, Mianyang City, Sichuan Province				2020.2.29-3.29	Chuanyu 18
20SMY_yt_4	Yanting County, Mianyang City, Sichuan Province				2020.2.29-3.29	Chuanmai 104
20SMY_yt_5	Yanting County, Mianyang City, Sichuan Province				2020.2.29-3.29	Chuanmai 104
20SMY_yt_6	Yanting County, Mianyang City, Sichuan Province				2020.2.29-3.29	Chuanmai 104
20SMY_zt_1	Chaiba Village, Changqing Town, Zitong, Mianyang City, Sichuan Province	31.3451	105.857	450	2020.1.13-2.3	Unknown
20SMY_zt_2	Chaiba Village, Changqing Town, Zitong, Mianyang City, Sichuan Province	31.3451	105.857	450	2020.1.13-2.3	Unknown
20SMY_zt_3	Chaiba Village, Changqing Town, Zitong, Mianyang City, Sichuan Province	31.3451	105.857	450	2020.1.13-2.3	Unknown

20SMY_zt_4	Chaiba Village, Changqing Town, Zitong, Mianyang City, Sichuan Province	31.3451	105.857	450	2020.1.13-2.3	Unknown
20SMY_zt_5	Chaiba Village, Changqing Town, Zitong, Mianyang City, Sichuan Province	31.3451	105.857	450	2020.1.13-2.3	Unknown
20SMY_zt_6	Chaiba Village, Changqing Town, Zitong, Mianyang City, Sichuan Province	31.3451	105.857	450	2020.1.13-2.3	Unknown
20SMY_zt_7	Chaiba Village, Changqing Town, Zitong, Mianyang City, Sichuan Province	31.3451	105.857	450	2020.1.13-2.3	Unknown
20SMY_zt_8	Chaiba Village, Changqing Town, Zitong, Mianyang City, Sichuan Province	31.3451	105.857	450	2020.1.13-2.3	Unknown
20SMY_zt_9	Chaiba Village, Changqing Town, Zitong, Mianyang City, Sichuan Province	31.3451	105.857	450	2020.1.13-2.3	Unknown
20SMY_zt_10	Chaiba Village, Changqing Town, Zitong, Mianyang City, Sichuan Province	31.3451	105.857	450	2020.1.13-2.3	Unknown
20SMY_zt_11	Linlong Village, Qibo Town, Yanting County, Mianyang City, Sichuan Province	31.461	105.331	502	2020.2.27-3.3	Unknown
20SMY_zt_12	Wenxing Town, Zitong County, Mianyang City, Sichuan Province	31.526	105.318	544	2020.2.27-3.3	Unknown
20SMY_zt_13	Wenxing Town, Zitong County, Mianyang City, Sichuan Province	31.526	105.318	544	2020.2.27-3.3	Unknown
20SMY_zt_14	Wenxing Town, Zitong County, Mianyang City, Sichuan Province	31.572	105.375	508	2020.2.27-3.3	Unknown
20SLS_hd_1	Xiaoba Town, Huidong County, Liangshan Yi Autonomous Prefecture, Sichuan Province	26.578985	102.2395096	1778	2020.1.13-1.14	Unknown
20SLS_hd_2	Xiaoba Town, Huidong County, Liangshan Yi Autonomous Prefecture, Sichuan Province	26.578985	102.2395096	1778	2020.1.13-1.14	Unknown
20SLS_hd_3	Xiaoba Town, Huidong County, Liangshan Yi Autonomous Prefecture, Sichuan Province	26.578985	102.2395096	1778	2020.1.13-1.14	Unknown
20SLS_hd_4	Xiaoba Town, Huidong County, Liangshan Yi Autonomous Prefecture, Sichuan Province	26.578985	102.2395096	1778	2020.1.13-1.14	Unknown
20SLS_hd_5	Xiaoba Town, Huidong County, Liangshan Yi Autonomous Prefecture, Sichuan Province	26.578985	102.2395096	1778	2020.1.13-1.14	Unknown
20SLS_hd_6	Xiaoba Town, Huidong County, Liangshan Yi Autonomous Prefecture, Sichuan Province	26.578985	102.2395096	1778	2020.1.13-1.14	Unknown
20SLS_hd_7	Xiaoba Town, Huidong County, Liangshan Yi Autonomous Prefecture, Sichuan Province	26.578985	102.2395096	1778	2020.1.13-1.14	Unknown

20SLS_hd_8	Xiaoba Town, Huidong County, Liangshan Yi Autonomous Prefecture, Sichuan Province	26.578985	102.2395096	1778	2020.1.13-1.14	Unknown
20SLS_hd_9	Xiaoba Town, Huidong County, Liangshan Yi Autonomous Prefecture, Sichuan Province	26.578985	102.2395096	1778	2020.1.13-1.14	Unknown
20SLS_hd_10	Xiaoba Town, Huidong County, Liangshan Yi Autonomous Prefecture, Sichuan Province	26.578985	102.2395096	1778	2020.1.13-1.14	Unknown
20SLS_hd_11	Xiaoba Town, Huidong County, Liangshan Yi Autonomous Prefecture, Sichuan Province	26.578985	102.2395096	1778	2020.1.13-1.14	Unknown
20SLS_nn_1	Heinigou Village, Jingxing Town, Ningnan County, Liangshan Yi Autonomous Prefecture, Sichuan Province	27.03766	102.76368	1008	2020.1.13-1.14	Unknown
20SLS_nn_2	Heinigou Village, Jingxing Town, Ningnan County, Liangshan Yi Autonomous Prefecture, Sichuan Province	27.03766	102.76368	1008	2020.1.13-1.14	Unknown
20SLS_nn_3	Heinigou Village, Jingxing Town, Ningnan County, Liangshan Yi Autonomous Prefecture, Sichuan Province	27.03766	102.76368	1008	2020.1.13-1.14	Unknown
20SLS_nn_4	Pisha Town, Ningnan County, Liangshan Yi Autonomous Prefecture, Sichuan Province	27.054207	102.745969	1008.2	2020.1.13-1.14	Unknown
20SLS_nn_5	Pisha Town, Ningnan County, Liangshan Yi Autonomous Prefecture, Sichuan Province	27.054207	102.745969	1008.2	2020.1.13-1.14	Unknown
20SLS_nn_6	Songxin Town, Ningnan County, Liangshan Yi Autonomous Prefecture, Sichuan Province	27.21571	102.61304	1120.8	2020.1.13-1.14	Unknown
20SLS_nn_7	Songxin Town, Ningnan County, Liangshan Yi Autonomous Prefecture, Sichuan Province	27.21571	102.61304	1120.8	2020.1.13-1.14	Unknown
20SLS_nn_8	Songxin Town, Ningnan County, Liangshan Yi Autonomous Prefecture, Sichuan Province	27.21571	102.61304	1120.8	2020.1.13-1.14	Unknown
20SLS_nn_9	Songxin Town, Ningnan County, Liangshan Yi Autonomous Prefecture, Sichuan Province	27.21571	102.61304	1120.8	2020.1.13-1.14	Unknown

20SLS_nn_10	Songxin Town, Ningnan County, Liangshan Yi Autonomous Prefecture, Sichuan Province	27.21571	102.61304	1120.8	2020.1.13-1.14	Unknown
20SLS_nn_11	Songxin Town, Ningnan County, Liangshan Yi Autonomous Prefecture, Sichuan Province	27.21571	102.61304	1120.8	2020.1.13-1.14	Unknown
20SLS_nn_12	Songxin Town, Ningnan County, Liangshan Yi Autonomous Prefecture, Sichuan Province	27.21571	102.61304	1120.8	2020.1.13-1.14	Unknown
20SLS_nn_13	Songxin Town, Ningnan County, Liangshan Yi Autonomous Prefecture, Sichuan Province	27.21571	102.61304	1120.8	2020.1.13-1.14	Unknown
20SLS_nn_14	Songxin Town, Ningnan County, Liangshan Yi Autonomous Prefecture, Sichuan Province	27.21571	102.61304	1120.8	2020.1.13-1.14	Unknown
20SLS_nn_15	Songxin Town, Ningnan County, Liangshan Yi Autonomous Prefecture, Sichuan Province	27.21571	102.61304	1120.8	2020.1.13-1.14	Unknown
20SLS_nn_16	Kamo Village, Jingxing Town, Ningnan County, Liangshan Yi Autonomous Prefecture, Sichuan Province	27.058033	102.75518	1024	2020.1.13-1.14	Unknown
20SLS_nn_17	Kamo Village, Jingxing Town, Ningnan County, Liangshan Yi Autonomous Prefecture, Sichuan Province	27.058033	102.75518	1024	2020.1.13-1.14	Unknown
20SLS_nn_18	Kamo Village, Jingxing Town, Ningnan County, Liangshan Yi Autonomous Prefecture, Sichuan Province	27.058033	102.75518	1024	2020.1.13-1.14	Unknown
20SLS_nn_19	Kamo Village, Jingxing Town, Ningnan County, Liangshan Yi Autonomous Prefecture, Sichuan Province	27.058033	102.75518	1024	2020.1.13-1.14	Unknown
20SLS_nn_20	Kamo Village, Jingxing Town, Ningnan County, Liangshan Yi Autonomous Prefecture, Sichuan Province	27.058033	102.75518	1024	2020.1.13-1.14	Unknown
20SLS_nn_21	Kamo Village, Jingxing Town, Ningnan County, Liangshan Yi Autonomous Prefecture, Sichuan Province	27.058033	102.75518	1024	2020.1.13-1.14	Unknown
20SLS_nn_22	Kamo Village, Jingxing Town, Ningnan County, Liangshan Yi Autonomous Prefecture, Sichuan Province	27.058033	102.75518	1024	2020.1.13-1.14	Unknown

20SLS_nn_23	Kamo Village, Jingxing Town, Ningnan County, Liangshan Yi Autonomous Prefecture, Sichuan Province	27.058033	102.75518	1024	2020.1.13-1.14	Unknown
20SLS_nn_24	Kamo Village, Jingxing Town, Ningnan County, Liangshan Yi Autonomous Prefecture, Sichuan Province	27.058033	102.75518	1024	2020.1.13-1.14	Unknown
20SLS_nn_25	Kamo Village, Jingxing Town, Ningnan County, Liangshan Yi Autonomous Prefecture, Sichuan Province	27.058033	102.75518	1024	2020.1.13-1.14	Unknown
20SLS_nn_26	Kamo Village, Jingxing Town, Ningnan County, Liangshan Yi Autonomous Prefecture, Sichuan Province	27.058033	102.75518	1024	2020.1.13-1.14	Unknown
20SLS_nn_27	Kamo Village, Jingxing Town, Ningnan County, Liangshan Yi Autonomous Prefecture, Sichuan Province	27.058033	102.75518	1024	2020.1.13-1.14	Unknown
20SLS_nn_28	Kamo Village, Jingxing Town, Ningnan County, Liangshan Yi Autonomous Prefecture, Sichuan Province	27.058033	102.75518	1024	2020.1.13-1.14	Unknown
20SLS_nn_29	Kamo Village, Jingxing Town, Ningnan County, Liangshan Yi Autonomous Prefecture, Sichuan Province	27.058033	102.75518	1024	2020.1.13-1.14	Unknown
20SLS_nn_30	Kamo Village, Jingxing Town, Ningnan County, Liangshan Yi Autonomous Prefecture, Sichuan Province	27.058033	102.75518	1024	2020.1.13-1.14	Unknown
20SLS_nn_31	Kamo Village, Jingxing Town, Ningnan County, Liangshan Yi Autonomous Prefecture, Sichuan Province	27.058033	102.75518	1024	2020.1.13-1.14	Unknown
20SLS_nn_32	Kamo Village, Jingxing Town, Ningnan County, Liangshan Yi Autonomous Prefecture, Sichuan Province	27.058033	102.75518	1024	2020.1.13-1.14	Unknown
20SLS_nn_33	Kamo Village, Jingxing Town, Ningnan County, Liangshan Yi Autonomous Prefecture, Sichuan Province	27.058033	102.75518	1024	2020.1.13-1.14	Unknown
20SLS_nn_34	Heinigou Village, Jingxing Town, Ningnan County, Liangshan Yi Autonomous Prefecture, Sichuan Province	27.03766	102.76368	1008	2020.1.13-1.14	Unknown
20SLS_nn_35	Heinigou Village, Jingxing Town, Ningnan County, Liangshan Yi Autonomous Prefecture, Sichuan Province	27.03766	102.76368	1008	2020.1.13-1.14	Unknown

20SLS_nn_36	Heinigou Village, Jingxing Town, Ningnan County, Liangshan Yi Autonomous Prefecture, Sichuan Province	27.03766	102.76368	1008	2020.1.13-1.14	Unknown
20SLS_nn_37	Heinigou Village, Jingxing Town, Ningnan County, Liangshan Yi Autonomous Prefecture, Sichuan Province	27.03766	102.76368	1008	2020.1.13-1.14	Unknown
20G_bj_1	Datian Village, Liuquhe Town, Hezhang County, Bijie City, Guizhou Province	27.230531	104.711843	1616.7	2020.1.9-1.12	Unknown
20G_bj_2	Datian Village, Liuquhe Town, Hezhang County, Bijie City, Guizhou Province	27.230531	104.711843	1616.7	2020.1.9-1.12	Unknown
20G_bj_3	Datian Village, Liuquhe Town, Hezhang County, Bijie City, Guizhou Province	27.230531	104.711843	1616.7	2020.1.9-1.12	Unknown
20G_lps_1	Gaoqiao Village, Yanjiao Town, Liupanshui City, Guizhou Province	26.35196	105.400954	1186	2020.1.9-1.12	Unknown
20G_lps_2	Gaoqiao Village, Yanjiao Town, Liupanshui City, Guizhou Province	26.35196	105.400954	1186	2020.1.9-1.12	Unknown
20G_gy_1	Huaxi District, Guiyang City, Guizhou Province	26.498147	106.589941	1112.5	2020.1.9-1.12	Unknown
20G_gy_2	Huaxi District, Guiyang City, Guizhou Province	26.498147	106.589941	1112.5	2020.1.9-1.12	Unknown
21SLZ_hj_1	Hejiang County, Luzhou City, Sichuan Province					Unknown
21SGY_jg_1	Youai Village, Kiafeng Town, Jiange County, Guangyuan City, Sichuan Province	31.8843	105.2824	650	2021.4.8-5.1	Unknown
21SGY_jg_2	Youai Village, Kiafeng Town, Jiange County, Guangyuan City, Sichuan Province	31.8843	105.2824	650	2021.4.8-5.1	Unknown
21SGY_jg_3	Youai Village, Kiafeng Town, Jiange County, Guangyuan City, Sichuan Province	31.8843	105.2824	650	2021.4.8-5.1	Unknown
21SGY_jg_4	Youai Village, Kiafeng Town, Jiange County, Guangyuan City, Sichuan Province	31.8843	105.2824	650	2021.4.8-5.1	Unknown
21SGY_jg_5	Youai Village, Kiafeng Town, Jiange County, Guangyuan City, Sichuan Province	31.8843	105.2824	650	2021.4.8-5.1	Unknown
21SGY_jg_6	Youai Village, Kiafeng Town, Jiange County, Guangyuan City, Sichuan Province	31.8843	105.2824	650	2021.4.8-5.1	Unknown
21SGY_jg_7	Youai Village, Kiafeng Town, Jiange County, Guangyuan City, Sichuan Province	31.8843	105.2824	650	2021.4.8-5.1	Unknown
21SGY_jg_8	Youai Village, Kiafeng Town, Jiange County, Guangyuan City, Sichuan Province	31.8843	105.2824	650	2021.4.8-5.1	Unknown
21SGY_jg_9	Youai Village, Kiafeng Town, Jiange County, Guangyuan City, Sichuan Province	31.8843	105.2824	650	2021.4.8-5.1	Unknown
21SGY_jg_10	Youai Village, Kiafeng Town, Jiange County, Guangyuan City, Sichuan Province	31.8843	105.2824	650	2021.4.8-5.1	Unknown
21SGY_jg_11	Youai Village, Kiafeng Town, Jiange County, Guangyuan City, Sichuan Province	31.8843	105.2824	650	2021.4.8-5.1	Unknown
21SMY_st_1	Duanshan Village, Le'an Town, Santai County, Mianyang City, Sichuan Province	31.0964	105.0145	359	2021.4.5-5.1	Unknown
21SMY_st_2	Duanshan Village, Le'an Town, Santai County, Mianyang City, Sichuan Province	31.0964	105.0145	359	2021.4.5-5.1	Unknown
21SMY_st_3	Duanshan Village, Le'an Town, Santai County, Mianyang City, Sichuan Province	31.0964	105.0145	359	2021.4.5-5.1	Unknown

21SMY_st_4	Duanshan Village, Le'an Town, Santai County, Mianyang City, Sichuan Province	31.1004	104.9911	392	2021.4.5-5.1	Unknown
21SMY_zt_1	Zitong County, Mianyang City, Sichuan Province					Unknown
21SMY_zt_2	Zitong County, Mianyang City, Sichuan Province					Unknown
21SMY_zt_3	Zitong County, Mianyang City, Sichuan Province					Unknown
21SMY_zt_4	Zitong County, Mianyang City, Sichuan Province					Unknown
21SMY_zt_5	Zitong County, Mianyang City, Sichuan Province					Unknown
21SMY_zt_6	Zitong County, Mianyang City, Sichuan Province					Unknown
21SMY_zt_7	Zitong County, Mianyang City, Sichuan Province					Unknown
21SMY_zt_8	Zitong County, Mianyang City, Sichuan Province					Unknown
21SMY_zt_9	Zitong County, Mianyang City, Sichuan Province					Unknown
21SMY_zt_10	Zitong County, Mianyang City, Sichuan Province					Unknown
21SMY_zt_11	Zitong County, Mianyang City, Sichuan Province					Unknown
21SMY_zt_12	Zitong County, Mianyang City, Sichuan Province					Unknown
21SMY_zt_13	Zitong County, Mianyang City, Sichuan Province					Unknown
21SDY_zj_1	Tongji Village, Tongji Town, Zhongjiang County, Deyang City, Sichuan Province	31.0817	104.7672	422	2021.4.11-5.2	Unknown
21SDY_zj_2	Tongji Village, Tongji Town, Zhongjiang County, Deyang City, Sichuan Province	31.0817	104.7672	422	2021.4.11-5.2	Unknown
21SDY_zj_3	Tongji Village, Tongji Town, Zhongjiang County, Deyang City, Sichuan Province	31.0817	104.7672	422	2021.4.11-5.2	Unknown
21SDY_zj_4	Tongji Village, Tongji Town, Zhongjiang County, Deyang City, Sichuan Province	31.0817	104.7672	422	2021.4.11-5.2	Unknown
21SDY_zj_5	Tongji Village, Tongji Town, Zhongjiang County, Deyang City, Sichuan Province	31.0817	104.7672	422	2021.4.11-5.2	Unknown
21SDY_zj_6	Tongji Village, Tongji Town, Zhongjiang County, Deyang City, Sichuan Province	31.0817	104.7672	422	2021.4.11-5.2	Unknown
21SDY_zj_7	Tongji Village, Tongji Town, Zhongjiang County, Deyang City, Sichuan Province	31.0817	104.7672	422	2021.4.11-5.2	Unknown
21SDY_zj_8	Tongji Village, Tongji Town, Zhongjiang County, Deyang City, Sichuan Province	31.0817	104.7672	422	2021.4.11-5.2	Unknown
21SLS_nn_1	Heinigou Village, Jingxing Town, Ningnan County, Liangshan Yi Autonomous Prefecture, Sichuan Province	27.03766	102.76368	1008	2021.2.3-3.3	Unknown
21SLS_nn_2	Heinigou Village, Jingxing Town, Ningnan County, Liangshan Yi Autonomous Prefecture, Sichuan Province	27.03766	102.76368	1008	2021.2.3-3.3	Unknown

21SLS_nn_3	Heinigou Village, Jingxing Town, Ningnan County, Liangshan Yi Autonomous Prefecture, Sichuan Province	27.03766	102.76368	1008	2021.2.3-3.3	Unknown
21SLS_nn_4	Heinigou Village, Jingxing Town, Ningnan County, Liangshan Yi Autonomous Prefecture, Sichuan Province	27.03766	102.76368	1008	2021.2.3-3.3	Unknown
21SLS_nn_5	Heinigou Village, Jingxing Town, Ningnan County, Liangshan Yi Autonomous Prefecture, Sichuan Province	27.03766	102.76368	1008	2021.2.3-3.3	Unknown
21SLS_nn_6	Heinigou Village, Jingxing Town, Ningnan County, Liangshan Yi Autonomous Prefecture, Sichuan Province	27.03766	102.76368	1008	2021.2.3-3.3	Unknown
21SLS_nn_7	Heinigou Village, Jingxing Town, Ningnan County, Liangshan Yi Autonomous Prefecture, Sichuan Province	27.03766	102.76368	1008	2021.2.3-3.3	Unknown
21SLS_nn_8	Xiacun Village, Pisha Town, Ningnan County, Liangshan Yi Autonomous Prefecture, Sichuan Province	27.0675	102.7346	1087	2021.2.3-3.3	Unknown
21SLS_nn_9	Sanchahe Village, Songxin Town, Ningnan County, Liangshan Yi Autonomous Prefecture, Sichuan Province	27.2136	102.609	940	2021.2.3-3.3	Unknown
21SLS_nn_10	Heinigou Village, Jingxing Town, Ningnan County, Liangshan Yi Autonomous Prefecture, Sichuan Province	27.03766	102.76368	1008	2021.3.23.-4.1	Unknown
21SLS_nn_11	Heinigou Village, Jingxing Town, Ningnan County, Liangshan Yi Autonomous Prefecture, Sichuan Province	27.03766	102.76368	1008	2021.3.23.-4.1	Unknown
21SLS_nn_12	Heinigou Village, Jingxing Town, Ningnan County, Liangshan Yi Autonomous Prefecture, Sichuan Province	27.03766	102.76368	1008	2021.3.23.-4.1	Unknown
21SLS_nn_13	Heinigou Village, Jingxing Town, Ningnan County, Liangshan Yi Autonomous Prefecture, Sichuan Province	27.03766	102.76368	1008	2021.3.23.-4.1	Unknown
21SLS_nn_14	Heinigou Village, Jingxing Town, Ningnan County, Liangshan Yi Autonomous Prefecture, Sichuan Province	27.03766	102.76368	1008	2021.3.23.-4.1	Unknown
21SLS_nn_15	Heinigou Village, Jingxing Town, Ningnan County, Liangshan Yi Autonomous Prefecture, Sichuan Province	27.03766	102.76368	1008	2021.3.23.-4.1	Unknown

21SLS_nn_16	Heinigou Village, Jingxing Town, Ningnan County, Liangshan Yi Autonomous Prefecture, Sichuan Province	27.03766	102.76368	1008	2021.3.23.-4.1	Unknown
21SLS_nn_17	Heinigou Village, Jingxing Town, Ningnan County, Liangshan Yi Autonomous Prefecture, Sichuan Province	27.03766	102.76368	1008	2021.3.23.-4.1	Unknown
21SLS_nn_18	Heinigou Village, Jingxing Town, Ningnan County, Liangshan Yi Autonomous Prefecture, Sichuan Province	27.03766	102.76368	1008	2021.3.23.-4.1	Mianyang 19
21SLS_nn_19	Heinigou Village, Jingxing Town, Ningnan County, Liangshan Yi Autonomous Prefecture, Sichuan Province	27.03766	102.76368	1008	2021.3.23.-4.1	Mianyang 19
21SLS_nn_20	Heinigou Village, Jingxing Town, Ningnan County, Liangshan Yi Autonomous Prefecture, Sichuan Province	27.03766	102.76368	1008	2021.3.23.-4.1	Mianyang 19
21SLS_nn_21	Heinigou Village, Jingxing Town, Ningnan County, Liangshan Yi Autonomous Prefecture, Sichuan Province	27.03766	102.76368	1008	2021.3.23.-4.1	Mianyang 19
21SLS_nn_22	Heinigou Village, Jingxing Town, Ningnan County, Liangshan Yi Autonomous Prefecture, Sichuan Province	27.03766	102.76368	1008	2021.3.23.-4.1	Mianyang 19
21SGZ_df_1	Bamei Town, Daofu County, Ganzi Tibetan Autonomous Prefecture, Sichuan Province	30.3219	101.2616	3440	2021.9.10-10.1	Longmai 4
21SGZ_df_2	Bamei Town, Daofu County, Ganzi Tibetan Autonomous Prefecture, Sichuan Province	30.3219	101.2616	3440	2021.9.10-10.1	Longmai 4
21SGZ_df_3	Bamei Town, Daofu County, Ganzi Tibetan Autonomous Prefecture, Sichuan Province	30.3219	101.2616	3440	2021.9.10-10.1	Longmai 4
21G_pz_1	Baotian Town, Panzhou City, Liupanshui City, Guizhou Province	25.4161	104.727	1549	2021.3.35-3.31	Abbondanza
21G_pz_2	Baotian Town, Panzhou City, Liupanshui City, Guizhou Province	25.4161	104.727	1549	2021.3.35-3.31	Abbondanza
21G_pz_3	Baotian Town, Panzhou City, Liupanshui City, Guizhou Province	25.4161	104.727	1549	2021.3.35-3.31	Abbondanza
21G_pz_4	Baotian Town, Panzhou City, Liupanshui City, Guizhou Province	25.4161	104.727	1549	2021.3.35-3.31	Abbondanza
21G_pz_5	Baotian Town, Panzhou City, Liupanshui City, Guizhou Province	25.4161	104.727	1549	2021.3.35-3.31	Abbondanza

21Y_cx_1	Duimen Village,Nanhua County, Chuxiong Yi Autonomous Prefecture, Yunnan Province	25.032	101.299	1830	2021.3.23-3.31	Unknown
21Y_cx_2	Duimen Village,Nanhua County, Chuxiong Yi Autonomous Prefecture, Yunnan Province	25.032	101.299	1830	2021.3.23-3.31	Unknown
21Y_cx_3	Duimen Village,Nanhua County, Chuxiong Yi Autonomous Prefecture, Yunnan Province	25.032	101.299	1830	2021.3.23-3.31	Unknown
21Y_cx_4	Duimen Village,Nanhua County, Chuxiong Yi Autonomous Prefecture, Yunnan Province	25.032	101.299	1830	2021.3.23-3.31	Unknown
21Y_cx_5	Duimen Village,Nanhua County, Chuxiong Yi Autonomous Prefecture, Yunnan Province	25.032	101.299	1830	2021.3.23-3.31	Unknown
21Y_cx_6	Duimen Village,Nanhua County, Chuxiong Yi Autonomous Prefecture, Yunnan Province	25.032	101.299	1830	2021.3.23-3.31	Unknown
21Y_cx_7	Duimen Village,Nanhua County, Chuxiong Yi Autonomous Prefecture, Yunnan Province	25.032	101.299	1830	2021.3.23-3.31	Unknown
21Y_cx_8	Duimen Village,Nanhua County, Chuxiong Yi Autonomous Prefecture, Yunnan Province	25.032	101.299	1830	2021.3.23-3.31	Unknown
21Y_cx_9	Duimen Village,Nanhua County, Chuxiong Yi Autonomous Prefecture, Yunnan Province	25.032	101.299	1830	2021.3.23-3.31	Unknown
21Y_cx_10	Duimen Village,Nanhua County, Chuxiong Yi Autonomous Prefecture, Yunnan Province	25.032	101.299	1830	2021.3.23-3.31	Unknown
21Y_cx_11	Duimen Village,Nanhua County, Chuxiong Yi Autonomous Prefecture, Yunnan Province	25.032	101.299	1830	2021.3.23-3.31	Unknown
21Y_cx_12	Duimen Village,Nanhua County, Chuxiong Yi Autonomous Prefecture, Yunnan Province	25.032	101.299	1830	2021.3.23-3.31	Unknown
21Y_cx_13	Duimen Village,Nanhua County, Chuxiong Yi Autonomous Prefecture, Yunnan Province	25.032	101.299	1830	2021.3.23-3.31	Unknown

21Y_cx_14	Duimen Village,Nanhua County, Chuxiong Yi Autonomous Prefecture, Yunnan Province	25.032	101.299	1830	2021.3.23-3.31	Unknown
21Y_cx_15	Duimen Village,Nanhua County, Chuxiong Yi Autonomous Prefecture, Yunnan Province	25.032	101.299	1830	2021.3.23-3.31	Unknown
21Y_qj_1	Duza Village, Xiongbi Town, Shizong County, Qujing City, Yunnan Province	24.8655	103.8032	2014	2021.3.24.-3.31	Unknown
21Y_qj_2	Duza Village, Xiongbi Town, Shizong County, Qujing City, Yunnan Province	24.8655	103.8032	2014	2021.3.24.-3.31	Unknown
21Y_qj_3	Duza Village, Xiongbi Town, Shizong County, Qujing City, Yunnan Province	24.8655	103.8032	2014	2021.3.24.-3.31	Unknown
21Y_qj_4	Duza Village, Xiongbi Town, Shizong County, Qujing City, Yunnan Province	24.8655	103.8032	2014	2021.3.24.-3.31	Unknown
21Y_qj_5	Duza Village, Xiongbi Town, Shizong County, Qujing City, Yunnan Province	24.8655	103.8032	2014	2021.3.24.-3.31	Unknown
21Y_qj_6	Duza Village, Xiongbi Town, Shizong County, Qujing City, Yunnan Province	24.8655	103.8032	2014	2021.3.24.-3.31	Unknown
21Y_qj_7	Duza Village, Xiongbi Town, Shizong County, Qujing City, Yunnan Province	24.8655	103.8032	2014	2021.3.24.-3.31	Unknown
21Y_qj_8	Duza Village, Xiongbi Town, Shizong County, Qujing City, Yunnan Province	24.8655	103.8032	2014	2021.3.24.-3.31	Unknown
21Y_qj_9	Duza Village, Xiongbi Town, Shizong County, Qujing City, Yunnan Province	24.8655	103.8032	2014	2021.3.24.-3.31	Unknown
21Y_qj_10	Duza Village, Xiongbi Town, Shizong County, Qujing City, Yunnan Province	24.8655	103.8032	2014	2021.3.24.-3.31	Unknown
21Y_qj_11	Duza Village, Xiongbi Town, Shizong County, Qujing City, Yunnan Province	24.8655	103.8032	2014	2021.3.24.-3.31	Unknown
21Y_qj_12	Luoxiong Street, Luoping County, Qujing City, Yunnan Province	24.5619	104.2129	1480	2021.3.24.-3.31	Unknown
21Y_hh_1	Zhongshan Village, Xunjiansi Town, Mile City, Qujing City, Honghe Hani and Yi Autonomous Prefecture, Yunnan Province	23.93	103.28	1443	2021.3.24.-3.31	Unknown
21Y_hh_2	Heifang Village, Xinshao Town, Mile City, Qujing City, Honghe Hani and Yi Autonomous Prefecture, Yunnan Province	24.31	103.35	1349	2021.3.24.-3.31	Unknown
21Y_hh_3	Hamie Village, Hongwo Town, Mile City, Qujing City, Honghe Hani and Yi Autonomous Prefecture, Yunnan Province	24.21	103.36	1398	2021.3.24.-3.31	Unknown
21Y_hh_4	Hamie Village, Hongwo Town, Mile City, Qujing City, Honghe Hani and Yi Autonomous Prefecture, Yunnan Province	24.21	103.36	1398	2021.3.24.-3.31	Unknown
21Y_hh_5	Hamie Village, Hongwo Town, Mile City, Qujing City, Honghe Hani and Yi Autonomous Prefecture, Yunnan Province	24.21	103.36	1398	2021.3.24.-3.31	Unknown
