# Supplementary material for: Differences in the Virulence Between Local Populations of Puccinia striiformis f. sp. tritici in Southwest China
Source: Plants (Basel). 2024 Oct 17;13(20):2902. doi: 10.3390/plants13202902 (PMC11511155; doi:10.3390/plants13202902)
Supplement: Supplementary file 1 [file plants-13-02902-s001.zip › Supplementary Table/Table S2.pdf]

Supplementary Table S2. The virulence and avirulence patterns of isolates (*Puccinia striiformis* f. sp. *tritici*) collected in 2020-2021 on the 21 differential genotypes.

**Strains**	***Pst* race or virulence type**	**1**	**2**	**3**	**4**	**5**	**6**	**7**	**8**	**9**	**10**	**11**	**12**	**13**	**14**	**15**	**16**	**17**	**18**	**19**	**20**	**21**
20SMY_yx_1	CYR34	S	S	S	S	S	S	S	S	S	S	S	S	S	S	R	S	S	R	S	S	S
20SMY_yx_2	CYR34	S	S	S	S	S	S	S	S	S	S	S	S	S	S	R	S	S	R	S	S	S
20SMY_yx_3	G22_014	S	S	S	S	S	S	S	S	S	S	S	S	S	S	R	S	R	R	S	S	S
20SMY_yx_4	CYR34	S	S	S	S	S	S	S	S	S	S	S	S	S	S	R	S	S	R	S	S	S
20SMY_yx_5	CYR34	S	S	S	S	S	S	S	S	S	S	S	S	S	S	R	S	S	R	S	S	S
20SMY_yx_6	CYR34	S	S	S	S	S	S	S	S	S	S	S	S	S	S	R	S	S	R	S	S	S
20SMY_jy_1	CYR34	S	S	S	S	S	S	S	S	S	S	S	S	S	S	R	S	S	R	S	S	S
20SMY_jy_2	CYR34	S	S	S	S	S	S	S	S	S	S	S	S	S	S	R	S	S	R	S	S	S
20SMY_jy_3	CYR34	S	S	S	S	S	S	S	S	S	S	S	S	S	S	R	S	S	R	S	S	S
20SMY_jy_4	CYR34	S	S	S	S	S	S	S	S	S	S	S	S	S	S	R	S	S	R	S	S	S
20SMY_jy_5	G22-244	S	S	S	S	S	S	S	S	S	S	S	S	S	S	R	R	R	R	S	S	S
20SMY_jy_6	CYR34	S	S	S	S	S	S	S	S	S	S	S	S	S	S	R	S	S	R	S	S	S
20SMY_jy_7	CYR34	S	S	S	S	S	S	S	S	S	S	S	S	S	S	R	S	S	R	S	S	S
20SMY_jy_8	CYR34	S	S	S	S	S	S	S	S	S	S	S	S	S	S	R	S	S	R	S	S	S
20SMY_jy_9	CYR34	S	S	S	S	S	S	S	S	S	S	S	S	S	S	R	S	S	R	S	S	S
20SMY_jy_10	G22_245	S	S	S	S	S	S	S	S	S	S	S	S	S	R	R	S	S	R	S	S	S
20SMY_jy_11	G22-244	S	S	S	S	S	S	S	S	S	S	S	S	S	S	R	R	R	R	S	S	S
20SGY_jg_1	G22-244	S	S	S	S	S	S	S	S	S	S	S	S	S	S	R	R	R	R	S	S	S
20SGY_jg_2	G22_183	S	S	S	S	S	S	S	S	S	R	S	S	R	S	R	S	S	R	S	S	S
20SGY_jg_3	G22_083	S	S	S	S	S	S	S	S	S	R	S	S	S	S	R	S	S	R	S	S	S
20SGY_jg_4	G22-244	S	S	S	S	S	S	S	S	S	S	S	S	S	S	R	R	R	R	S	S	S
20SGY_jg_5	CYR34	S	S	S	S	S	S	S	S	S	S	S	S	S	S	R	S	S	R	S	S	S
20SGY_jg_6	CYR34	S	S	S	S	S	S	S	S	S	S	S	S	S	S	R	S	S	R	S	S	S
20SMY_yt_1	CYR34	S	S	S	S	S	S	S	S	S	S	S	S	S	S	R	S	S	R	S	S	S
20SMY_yt_2	G22_104	S	S	S	S	S	S	S	S	S	S	S	S	S	S	R	R	S	R	S	S	S
20SMY_yt_3	G22-244	S	S	S	S	S	S	S	S	S	S	S	S	S	S	R	R	R	R	S	S	S
20SMY_yt_4	G22_104	S	S	S	S	S	S	S	S	S	S	S	S	S	S	R	R	S	R	S	S	S
20SMY_yt_5	CYR34	S	S	S	S	S	S	S	S	S	S	S	S	S	S	R	S	S	R	S	S	S
20SMY_yt_6	G22_104	S	S	S	S	S	S	S	S	S	S	S	S	S	S	R	R	S	R	S	S	S
20SMY_zt_1	CYR34	S	S	S	S	S	S	S	S	S	S	S	S	S	S	R	S	S	R	S	S	S
20SMY_zt_2	G22_104	S	S	S	S	S	S	S	S	S	S	S	S	S	S	R	R	S	R	S	S	S
20SMY_zt_3	CYR34	S	S	S	S	S	S	S	S	S	S	S	S	S	S	R	S	S	R	S	S	S
20SMY_zt_4	G22_104	S	S	S	S	S	S	S	S	S	S	S	S	S	S	R	R	S	R	S	S	S
20SMY_zt_5	CYR34	S	S	S	S	S	S	S	S	S	S	S	S	S	S	R	S	S	R	S	S	S
20SMY_zt_6	CYR34	S	S	S	S	S	S	S	S	S	S	S	S	S	S	R	S	S	R	S	S	S
20SMY_zt_7	CYR34	S	S	S	S	S	S	S	S	S	S	S	S	S	S	R	S	S	R	S	S	S

20SMY_zt_8	CYR34	S	S	S	S	S	S	S	S	S	S	S	S	S	S	R	S	S	R	S	S	S
20SMY_zt_9	CYR34	S	S	S	S	S	S	S	S	S	S	S	S	S	S	R	S	S	R	S	S	S
20SMY_zt_10	CYR34	S	S	S	S	S	S	S	S	S	S	S	S	S	S	R	S	S	R	S	S	S
20SMY_zt_11	CYR34	S	S	S	S	S	S	S	S	S	S	S	S	S	S	R	S	S	R	S	S	S
20SMY_zt_12	CYR34	S	S	S	S	S	S	S	S	S	S	S	S	S	S	R	S	S	R	S	S	S
20SMY_zt_13	CYR34	S	S	S	S	S	S	S	S	S	S	S	S	S	S	R	S	S	R	S	S	S
20SMY_zt_14	CYR34	S	S	S	S	S	S	S	S	S	S	S	S	S	S	R	S	S	R	S	S	S
20SLS_hd_1	CYR32	S	S	S	S	S	S	S	S	S	S	S	S	S	S	R	S	S	R	R	S	S
20SLS_hd_2	CYR32	S	S	S	S	S	S	S	S	S	S	S	S	S	S	R	S	S	R	R	S	S
20SLS_hd_3	CYR32	S	S	S	S	S	S	S	S	S	S	S	S	S	S	R	S	S	R	R	S	S
20SLS_hd_4	CYR32	S	S	S	S	S	S	S	S	S	S	S	S	S	S	R	S	S	R	R	S	S
20SLS_hd_5	CYR32	S	S	S	S	S	S	S	S	S	S	S	S	S	S	R	S	S	R	R	S	S
20SLS_hd_6	CYR32	S	S	S	S	S	S	S	S	S	S	S	S	S	S	R	S	S	R	R	S	S
20SLS_hd_8	CYR32	S	S	S	S	S	S	S	S	S	S	S	S	S	S	R	S	S	R	R	S	S
20SLS_hd_9	CYR32	S	S	S	S	S	S	S	S	S	S	S	S	S	S	R	S	S	R	R	S	S
20SLS_hd_10	CYR32	S	S	S	S	S	S	S	S	S	S	S	S	S	S	R	S	S	R	R	S	S
20SLS_hd_11	CYR32	S	S	S	S	S	S	S	S	S	S	S	S	S	S	R	S	S	R	R	S	S
20SLS_nn_1	CYR32	S	S	S	S	S	S	S	S	S	S	S	S	S	S	R	S	S	R	R	S	S
20SLS_nn_2	CYR32	S	S	S	S	S	S	S	S	S	S	S	S	S	S	R	S	S	R	R	S	S
20SLS_nn_3	CYR32	S	S	S	S	S	S	S	S	S	S	S	S	S	S	R	S	S	R	R	S	S
20SLS_nn_4	CYR32	S	S	S	S	S	S	S	S	S	S	S	S	S	S	R	S	S	R	R	S	S
20SLS_nn_5	CYR32	S	S	S	S	S	S	S	S	S	S	S	S	S	S	R	S	S	R	R	S	S
20SLS_nn_6	CYR32	S	S	S	S	S	S	S	S	S	S	S	S	S	S	R	S	S	R	R	S	S
20SLS_nn_7	CYR32	S	S	S	S	S	S	S	S	S	S	S	S	S	S	R	S	S	R	R	S	S
20SLS_nn_8	CYR32	S	S	S	S	S	S	S	S	S	S	S	S	S	S	R	S	S	R	R	S	S
20SLS_nn_9	CYR32	S	S	S	S	S	S	S	S	S	S	S	S	S	S	R	S	S	R	R	S	S
20SLS_nn_10	CYR32	S	S	S	S	S	S	S	S	S	S	S	S	S	S	R	S	S	R	R	S	S
20SLS_nn_11	CYR32	S	S	S	S	S	S	S	S	S	S	S	S	S	S	R	S	S	R	R	S	S
20SLS_nn_12	CYR32	S	S	S	S	S	S	S	S	S	S	S	S	S	S	R	S	S	R	R	S	S
20SLS_nn_13	CYR32	S	S	S	S	S	S	S	S	S	S	S	S	S	S	R	S	S	R	R	S	S
20SLS_nn_14	CYR32	S	S	S	S	S	S	S	S	S	S	S	S	S	S	R	S	S	R	R	S	S

20SLS_nn_15	CYR32	S	S	S	S	S	S	S	S	S	S	S	S	S	S	R	S	S	R	R	S	S
20SLS_nn_16	CYR32	S	S	S	S	S	S	S	S	S	S	S	S	S	S	R	S	S	R	R	S	S
20SLS_nn_17	CYR32	S	S	S	S	S	S	S	S	S	S	S	S	S	S	R	S	S	R	R	S	S
20SLS_nn_18	CYR32	S	S	S	S	S	S	S	S	S	S	S	S	S	S	R	S	S	R	R	S	S
20SLS_nn_19	CYR32	S	S	S	S	S	S	S	S	S	S	S	S	S	S	R	S	S	R	R	S	S
20SLS_nn_20	CYR32	S	S	S	S	S	S	S	S	S	S	S	S	S	S	R	S	S	R	R	S	S
20SLS_nn_21	CYR32	S	S	S	S	S	S	S	S	S	S	S	S	S	S	R	S	S	R	R	S	S
20SLS_nn_22	CYR32	S	S	S	S	S	S	S	S	S	S	S	S	S	S	R	S	S	R	R	S	S
20SLS_nn_23	CYR32	S	S	S	S	S	S	S	S	S	S	S	S	S	S	R	S	S	R	R	S	S
20SLS_nn_24	CYR32	S	S	S	S	S	S	S	S	S	S	S	S	S	S	R	S	S	R	R	S	S
20SLS_nn_25	CYR32	S	S	S	S	S	S	S	S	S	S	S	S	S	S	R	S	S	R	R	S	S
20SLS_nn_26	CYR32	S	S	S	S	S	S	S	S	S	S	S	S	S	S	R	S	S	R	R	S	S
20SLS_nn_27	CYR32	S	S	S	S	S	S	S	S	S	S	S	S	S	S	R	S	S	R	R	S	S
20SLS_nn_28	CYR32	S	S	S	S	S	S	S	S	S	S	S	S	S	S	R	S	S	R	R	S	S
20SLS_nn_29	CYR32	S	S	S	S	S	S	S	S	S	S	S	S	S	S	R	S	S	R	R	S	S
20SLS_nn_30	CYR32	S	S	S	S	S	S	S	S	S	S	S	S	S	S	R	S	S	R	R	S	S
20SLS_nn_31	CYR32	S	S	S	S	S	S	S	S	S	S	S	S	S	S	R	S	S	R	R	S	S
20SLS_nn_32	CYR32	S	S	S	S	S	S	S	S	S	S	S	S	S	S	R	S	S	R	R	S	S
20SLS_nn_33	CYR32	S	S	S	S	S	S	S	S	S	S	S	S	S	S	R	S	S	R	R	S	S
20SLS_nn_34	CYR34	S	S	S	S	S	S	S	S	S	S	S	S	S	S	R	S	S	R	S	S	S
20SLS_nn_35	CYR32	S	S	S	S	S	S	S	S	S	S	S	S	S	S	R	S	S	R	R	S	S
20SLS_nn_36	CYR32	S	S	S	S	S	S	S	S	S	S	S	S	S	S	R	S	S	R	R	S	S

20SLS_nn_37	CYR34	S	S	S	S	S	S	S	S	S	S	S	S	S	S	R	S	S	R	S	S	S
20G_bj_1	CYR32	S	S	S	S	S	S	S	S	S	S	S	S	S	S	R	S	S	R	R	S	S
20G_bj_2	HY_108	S	S	S	S	S	S	S	S	S	S	S	S	S	S	R	R	S	R	R	S	S
20G_bj_3	HY_108	S	S	S	S	S	S	S	S	S	S	S	S	S	S	R	R	S	R	R	S	S
20G_lps_1	HY_108	S	S	S	S	S	S	S	S	S	S	S	S	S	S	R	R	S	R	R	S	S
20G_lps_2	CYR32	S	S	S	S	S	S	S	S	S	S	S	S	S	S	R	S	S	R	R	S	S
20G_gy_1	CYR32	S	S	S	S	S	S	S	S	S	S	S	S	S	S	R	S	S	R	R	S	S
20G_gy_2	CYR32	S	S	S	S	S	S	S	S	S	S	S	S	S	S	R	S	S	R	R	S	S
21SLZ_hj_1	G22_108	S	S	S	S	S	S	S	S	S	S	S	S	R	S	R	S	S	R	S	S	S
21SGY_jg_1	CYR34	S	S	S	S	S	S	S	S	S	S	S	S	S	S	R	S	S	R	S	S	S
21SGY_jg_2	CYR34	S	S	S	S	S	S	S	S	S	S	S	S	S	S	R	S	S	R	S	S	S
21SGY_jg_3	CYR34	S	S	S	S	S	S	S	S	S	S	S	S	S	S	R	S	S	R	S	S	S
21SGY_jg_4	CYR34	S	S	S	S	S	S	S	S	S	S	S	S	S	S	R	S	S	R	S	S	S
21SGY_jg_5	CYR34	S	S	S	S	S	S	S	S	S	S	S	S	S	S	R	S	S	R	S	S	S
21SGY_jg_6	CYR34	S	S	S	S	S	S	S	S	S	S	S	S	S	S	R	S	S	R	S	S	S
21SGY_jg_7	CYR34	S	S	S	S	S	S	S	S	S	S	S	S	S	S	R	S	S	R	S	S	S
21SGY_jg_8	CYR34	S	S	S	S	S	S	S	S	S	S	S	S	S	S	R	S	S	R	S	S	S
21SGY_jg_9	G22_108	S	S	S	S	S	S	S	S	S	S	S	S	R	S	R	S	S	R	S	S	S
21SGY_jg_10	CYR34	S	S	S	S	S	S	S	S	S	S	S	S	S	S	R	S	S	R	S	S	S
21SGY_jg_11	CYR34	S	S	S	S	S	S	S	S	S	S	S	S	S	S	R	S	S	R	S	S	S
21SMY_st_1	CYR34	S	S	S	S	S	S	S	S	S	S	S	S	S	S	R	S	S	R	S	S	S
21SMY_st_2	CYR34	S	S	S	S	S	S	S	S	S	S	S	S	S	S	R	S	S	R	S	S	S
21SMY_st_3	CYR34	S	S	S	S	S	S	S	S	S	S	S	S	S	S	R	S	S	R	S	S	S
21SMY_st_4	G22_183	S	S	S	S	S	S	S	S	S	R	S	S	R	S	R	S	S	R	S	S	S
21SMY_zt_1	CYR34	S	S	S	S	S	S	S	S	S	S	S	S	S	S	R	S	S	R	S	S	S
21SMY_zt_2	CYR34	S	S	S	S	S	S	S	S	S	S	S	S	S	S	R	S	S	R	S	S	S
21SMY_zt_3	G22_108	S	S	S	S	S	S	S	S	S	S	S	S	R	S	R	S	S	R	S	S	S
21SMY_zt_4	CYR34	S	S	S	S	S	S	S	S	S	S	S	S	S	S	R	S	S	R	S	S	S
21SMY_zt_5	CYR34	S	S	S	S	S	S	S	S	S	S	S	S	S	S	R	S	S	R	S	S	S
21SMY_zt_6	CYR34	S	S	S	S	S	S	S	S	S	S	S	S	S	S	R	S	S	R	S	S	S
21SMY_zt_7	CYR34	S	S	S	S	S	S	S	S	S	S	S	S	S	S	R	S	S	R	S	S	S
21SMY_zt_8	CYR34	S	S	S	S	S	S	S	S	S	S	S	S	S	S	R	S	S	R	S	S	S
21SMY_zt_9	CYR34	S	S	S	S	S	S	S	S	S	S	S	S	S	S	R	S	S	R	S	S	S
21SMY_zt_10	CYR34	S	S	S	S	S	S	S	S	S	S	S	S	S	S	R	S	S	R	S	S	S
21SMY_zt_11	CYR34	S	S	S	S	S	S	S	S	S	S	S	S	S	S	R	S	S	R	S	S	S
21SMY_zt_12	CYR34	S	S	S	S	S	S	S	S	S	S	S	S	S	S	R	S	S	R	S	S	S
21SMY_zt_13	CYR34	S	S	S	S	S	S	S	S	S	S	S	S	S	S	R	S	S	R	S	S	S
21SDY_zj_1	Other G22_1	R	S	S	S	S	S	S	S	S	R	S	S	R	S	R	S	S	R	S	S	S

21SDY_zj_2	G22_108	S	S	S	S	S	S	S	S	S	S	S	S	R	S	R	S	S	R	S	S	S
21SDY_zj_3	Other G22_1	R	S	S	S	S	S	S	S	S	R	S	S	R	S	R	S	S	R	S	S	S
21SDY_zj_4	G22_083	S	S	S	S	S	S	S	S	S	R	S	S	S	S	R	S	S	R	S	S	S
21SDY_zj_5	Other G22_1	R	S	S	S	S	S	S	S	S	R	S	S	R	S	R	S	S	R	S	S	S
21SDY_zj_6	Other G22_1	R	S	S	S	S	S	S	S	S	R	S	S	R	S	R	S	S	R	S	S	S
21SDY_zj_7	G22_083	S	S	S	S	S	S	S	S	S	R	S	S	S	S	R	S	S	R	S	S	S
21SDY_zj_8	G22_431	R	S	S	S	S	S	S	S	S	R	S	S	S	S	R	S	S	R	S	S	S
21SLS_nn_1	HY_183	S	S	S	S	S	S	S	S	S	R	S	R	R	S	R	S	S	R	R	S	S
21SLS_nn_2	Other HY46_1	S	S	S	S	S	S	S	S	R	R	S	S	R	S	R	S	S	R	R	S	S
21SLS_nn_3	CYR32	S	S	S	S	S	S	S	S	S	S	S	S	S	S	R	S	S	R	R	S	S
21SLS_nn_4	HY_311	S	S	R	S	S	S	S	S	S	R	S	S	R	S	R	S	S	R	R	S	S
21SLS_nn_5	HY_311	S	S	R	S	S	S	S	S	S	R	S	S	R	S	R	S	S	R	R	S	S
21SLS_nn_6	HY_008_1	S	S	S	S	S	S	S	S	S	S	S	S	R	S	R	S	S	R	R	S	S
21SLS_nn_7	Other HY46_2	S	S	R	S	S	S	S	S	S	R	S	S	R	S	R	R	S	R	R	S	S
21SLS_nn_8	HY_019	S	S	S	S	S	S	S	S	S	R	S	S	S	S	R	S	S	R	R	S	S
21SLS_nn_9	HY_008_1	S	S	S	S	S	S	S	S	S	S	S	S	R	S	R	S	S	R	R	S	S
21SLS_nn_10	CYR31	S	S	S	S	S	S	S	S	S	R	S	S	R	S	R	S	S	R	R	S	S
21SLS_nn_11	CYR32	S	S	S	S	S	S	S	S	S	S	S	S	S	S	R	S	S	R	R	S	S
21SLS_nn_12	CYR32	S	S	S	S	S	S	S	S	S	S	S	S	S	S	R	S	S	R	R	S	S
21SLS_nn_13	CYR32	S	S	S	S	S	S	S	S	S	S	S	S	S	S	R	S	S	R	R	S	S
21SLS_nn_14	CYR31	S	S	S	S	S	S	S	S	S	R	S	S	R	S	R	S	S	R	R	S	S
21SLS_nn_15	CYR31	S	S	S	S	S	S	S	S	S	R	S	S	R	S	R	S	S	R	R	S	S
21SLS_nn_16	HY_311	S	S	R	S	S	S	S	S	S	R	S	S	R	S	R	S	S	R	R	S	S
21SLS_nn_17	HY_008_1	S	S	S	S	S	S	S	S	S	S	S	S	R	S	R	S	S	R	R	S	S
21SLS_nn_18	CYR31	S	S	S	S	S	S	S	S	S	R	S	S	R	S	R	S	S	R	R	S	S
21SLS_nn_19	HY_019	S	S	S	S	S	S	S	S	S	R	S	S	S	S	R	S	S	R	R	S	S

21SLS_nn_20	CYR31	S	S	S	S	S	S	S	S	S	R	S	S	R	S	R	S	S	R	R	S	S
21SLS_nn_21	HY_008_1	S	S	S	S	S	S	S	S	S	S	S	S	R	S	R	S	S	R	R	S	S
21SLS_nn_22	CYR31	S	S	S	S	S	S	S	S	S	R	S	S	R	S	R	S	S	R	R	S	S
21SGZ_df_1	CYR34	S	S	S	S	S	S	S	S	S	S	S	S	S	S	R	S	S	R	S	S	S
21SGZ_df_2	CYR34	S	S	S	S	S	S	S	S	S	S	S	S	S	S	R	S	S	R	S	S	S
21SGZ_df_3	CYR34	S	S	S	S	S	S	S	S	S	S	S	S	S	S	R	S	S	R	S	S	S
21G_pz_1	CYR31	S	S	S	S	S	S	S	S	S	R	S	S	R	S	R	S	S	R	R	S	S
21G_pz_2	CYR32	S	S	S	S	S	S	S	S	S	S	S	S	S	S	R	S	S	R	R	S	S
21G_pz_3	CYR32	S	S	S	S	S	S	S	S	S	S	S	S	S	S	R	S	S	R	R	S	S
21G_pz_4	Other HY46_3	R	R	R	S	S	S	S	S	S	R	S	R	R	S	R	S	S	R	R	S	S
21G_pz_5	CYR31	S	S	S	S	S	S	S	S	S	R	S	S	R	S	R	S	S	R	R	S	S
21Y_cx_1	CYR32	S	S	S	S	S	S	S	S	S	S	S	S	S	S	R	S	S	R	R	S	R
21Y_cx_2	CYR32	S	S	S	S	S	S	S	S	S	S	S	S	S	S	R	S	S	R	R	S	R
21Y_cx_3	CYR32	S	S	S	S	S	S	S	S	S	S	S	S	S	S	R	S	S	R	R	S	R
21Y_cx_4	CYR32	S	S	S	S	S	S	S	S	S	S	S	S	S	S	R	S	S	R	R	S	R
21Y_cx_5	CYR32	S	S	S	S	S	S	S	S	S	S	S	S	S	S	R	S	S	R	R	S	R
21Y_cx_6	CYR32	S	S	S	S	S	S	S	S	S	S	S	S	S	S	R	S	S	R	R	S	R
21Y_cx_7	CYR32	S	S	S	S	S	S	S	S	S	S	S	S	S	S	R	S	S	R	R	S	R
21Y_cx_8	CYR32	S	S	S	S	S	S	S	S	S	S	S	S	S	S	R	S	S	R	R	S	R
21Y_cx_9	CYR32	S	S	S	S	S	S	S	S	S	S	S	S	S	S	R	S	S	R	R	S	R
21Y_cx_10	CYR32	S	S	S	S	S	S	S	S	S	S	S	S	S	S	R	S	S	R	R	S	R
21Y_cx_11	HY_035	R	S	S	S	S	S	S	S	S	R	S	S	R	S	R	S	S	R	R	S	R
21Y_cx_12	CYR32	S	S	S	S	S	S	S	S	S	S	S	S	S	S	R	S	S	R	R	S	R
21Y_cx_13	CYR32	S	S	S	S	S	S	S	S	S	S	S	S	S	S	R	S	S	R	R	S	R
21Y_cx_14	CYR32	S	S	S	S	S	S	S	S	S	S	S	S	S	S	R	S	S	R	R	S	R
21Y_cx_15	CYR32	S	S	S	S	S	S	S	S	S	S	S	S	S	S	R	S	S	R	R	S	R
21Y_qj_1	CYR32	S	S	S	S	S	S	S	S	S	S	S	S	S	S	R	S	S	R	R	S	R
21Y_qj_2	CYR32	S	S	S	S	S	S	S	S	S	S	S	S	S	S	R	S	S	R	R	S	R
21Y_qj_3	CYR32	S	S	S	S	S	S	S	S	S	S	S	S	S	S	R	S	S	R	R	S	R
21Y_qj_4	CYR32	S	S	S	S	S	S	S	S	S	S	S	S	S	S	R	S	S	R	R	S	R
21Y_qj_5	CYR32	S	S	S	S	S	S	S	S	S	S	S	S	S	S	R	S	S	R	R	S	R
21Y_qj_6	CYR32	S	S	S	S	S	S	S	S	S	S	S	S	S	S	R	S	S	R	R	S	R
21Y_qj_7	CYR32	S	S	S	S	S	S	S	S	S	S	S	S	S	S	R	S	S	R	R	S	R
21Y_qj_8	CYR32	S	S	S	S	S	S	S	S	S	S	S	S	S	S	R	S	S	R	R	S	R
21Y_qj_9	CYR32	S	S	S	S	S	S	S	S	S	S	S	S	S	S	R	S	S	R	R	S	R
21Y_qj_10	CYR32	S	S	S	S	S	S	S	S	S	S	S	S	S	S	R	S	S	R	R	S	R
21Y_qj_11	CYR32	S	S	S	S	S	S	S	S	S	S	S	S	S	S	R	S	S	R	R	S	R
21Y_qj_12	Other HY46_4	S	S	R	S	S	S	S	S	S	R	S	S	S	S	R	S	S	R	R	S	R
21Y_hh_1	CYR32	S	S	S	S	S	S	S	S	S	S	S	S	S	S	R	S	S	R	R	S	R

21Y_hh_2	CYR32	S	S	S	S	S	S	S	S	S	S	S	S	S	S	R	S	S	R	R	S	R
21Y_hh_3	CYR32	S	S	S	S	S	S	S	S	S	S	S	S	S	S	R	S	S	R	R	S	R
21Y_hh_4	CYR32	S	S	S	S	S	S	S	S	S	S	S	S	S	S	R	S	S	R	R	S	R
21Y_hh_5	CYR32	S	S	S	S	S	S	S	S	S	S	S	S	S	S	R	S	S	R	R	S	R

* 1-21 are differential wheat genotypes; 1, Trigo_Eureka; 2, Fulhard; 3, Lutescens 128; 4, Mentana; 5, Virgilio; 6, Abbondanza; 7, Early_Premium; 8, Funo; 9, Danish_1; 10, Jubilejina_2; 11, Fengchan 3; 12, Lovrin 13; 13, Kangyin_655; 14, Suwon_11; 15, Zhong_4; 16, Lovrin_10; 17, Hybrid_46; 18, *Triticum spelta album;* 19, Guinong 22; 20, MingXian 169; 21, Chuanmai 104. Chuanmai 104 was used as supplementary differential genotype in Sichuan. Infection types 0-2 were recorded as "R", and infection types 3-4 were was recorded as "S". “Other G22”, other unnamed “G22” group; “Other HY46”, other unnamed “Hybrid 46” group.
